# Supplementary material for: Signatures of omicron-like adaptation in early SARS-CoV-2 variants and chronic infection
Source: Cell Rep. Author manuscript; Available in PMC 2025 Dec 18. (PMC7618492; doi:10.1016/j.celrep.2025.116135)
Supplement: Supplementary Material [file EMS211251-supplement-Supplementary_Material.zip › 1-s2.0-S2211124725009064-mmc1.pdf]

Cell Reports, Volume *44*

Supplemental information

Signatures of omicron-like adaptation

in early SARS-CoV-2 variants and chronic infection

Mark Tsz Kin Cheng, Mazharul Altaf, Jesu Castin, Ann-Kathrin Reuschl, Benjamin L. Sievers, Kimia Kamelian, Dejan Mesner, Rebecca B. Morse, Adam Abdullahi, Bo Meng, Kata Csiba, Cambridge NIHR Bioresource, Steven A. Kemp, Darren P. Martin, Clare Jolly, Christopher Ruis, Lipi Thukral, and Ravindra K. Gupta

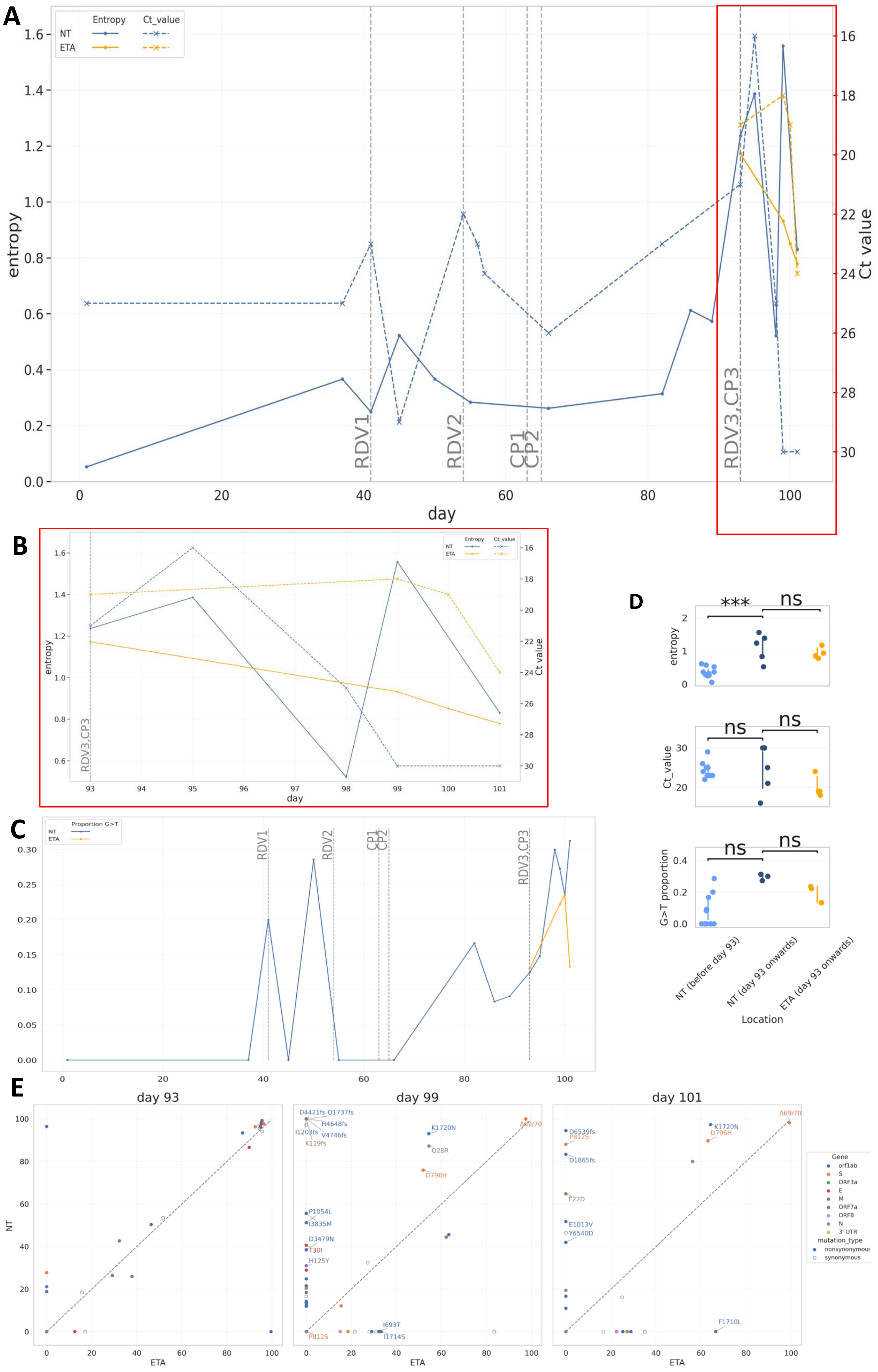

Supplementary Figure 1: Viral load and Intra-host evolution dynamics. A) Shannon's entropy (solid line, left y-axis) and Ct value (dotted line, right y-axis) of nose and throat (N+T, blue) and endotracheal aspirate (ETA, yellow) samples, zoomed in and rescaled from day 93 to day 101 (B). CP, convalescent plasma; RDV, Remdesivir. C) proportion of G to T mutation from day 1 to day 101. Day 93 and Day 95 were excluded as they are separated from the main clade. D) Dot plot (with 95% confidence interval) of Shannon's entropy (top) and Ct value (bottom) for NT samples from day 1-92, NT samples from day 93-101, and ETA samples from day 93 to 101. Student's t-test is performed for entropy whilst Welch correction was performed for Ct value in lieu of unequal variance. E) The percentage prevalence in NT plotted against ETA. Nonsynonymous mutations (filled) with >30% difference are annotated. The grey reference line represents equivalent prevalence between NT and ETA.

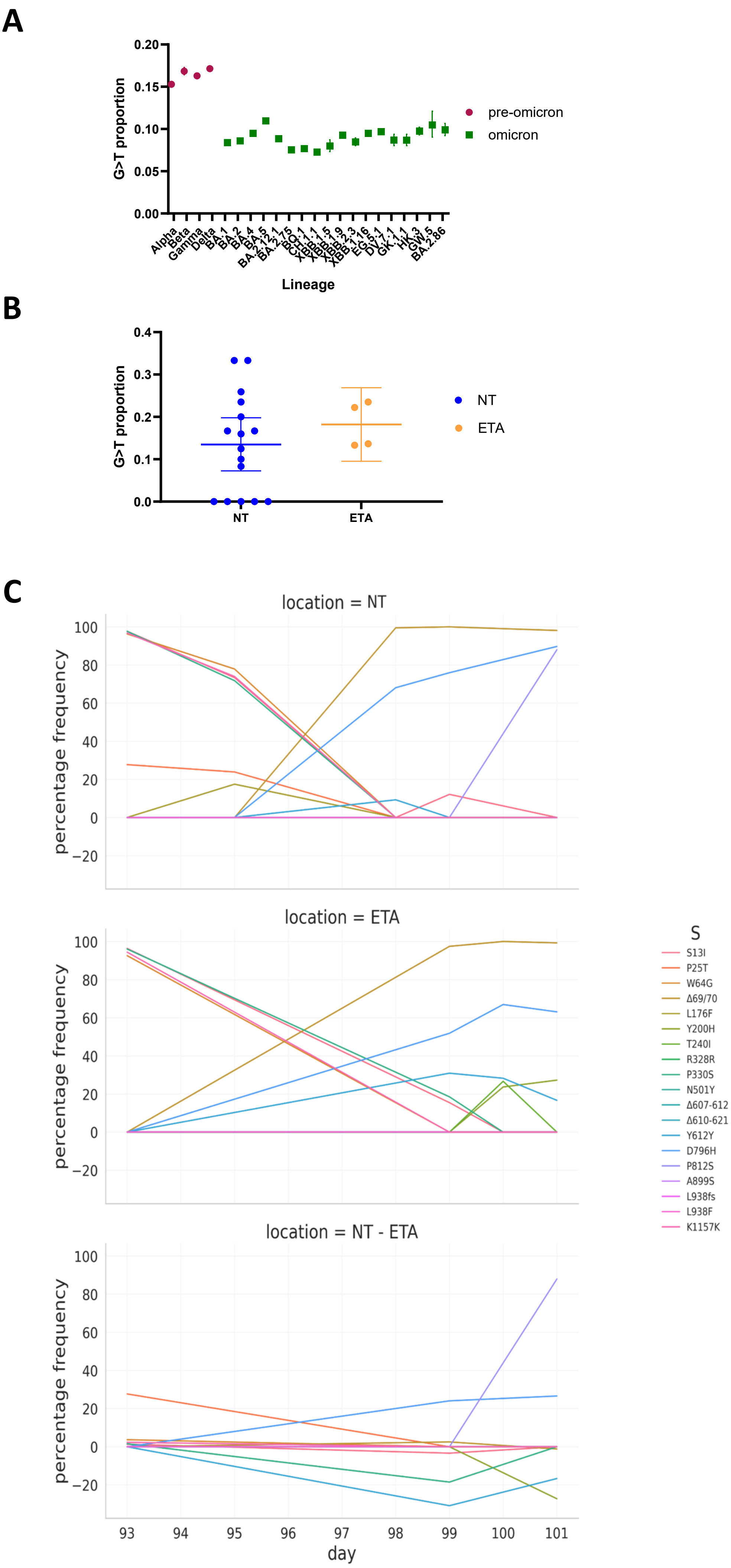

Supplementary Figure 2: Breakdown of mutational spectra and viral iSNV dynamics in the nasal and endotracheal compartments. The G to T proportion of A) globally circulating lineages in chronological order and B) of patient sample broken down by NT and ETA samples across all timepoints. C) Spike mutation frequency in N+T (top), ETA (middle), and absolute percentage difference (bottom) between day 93 and 101.

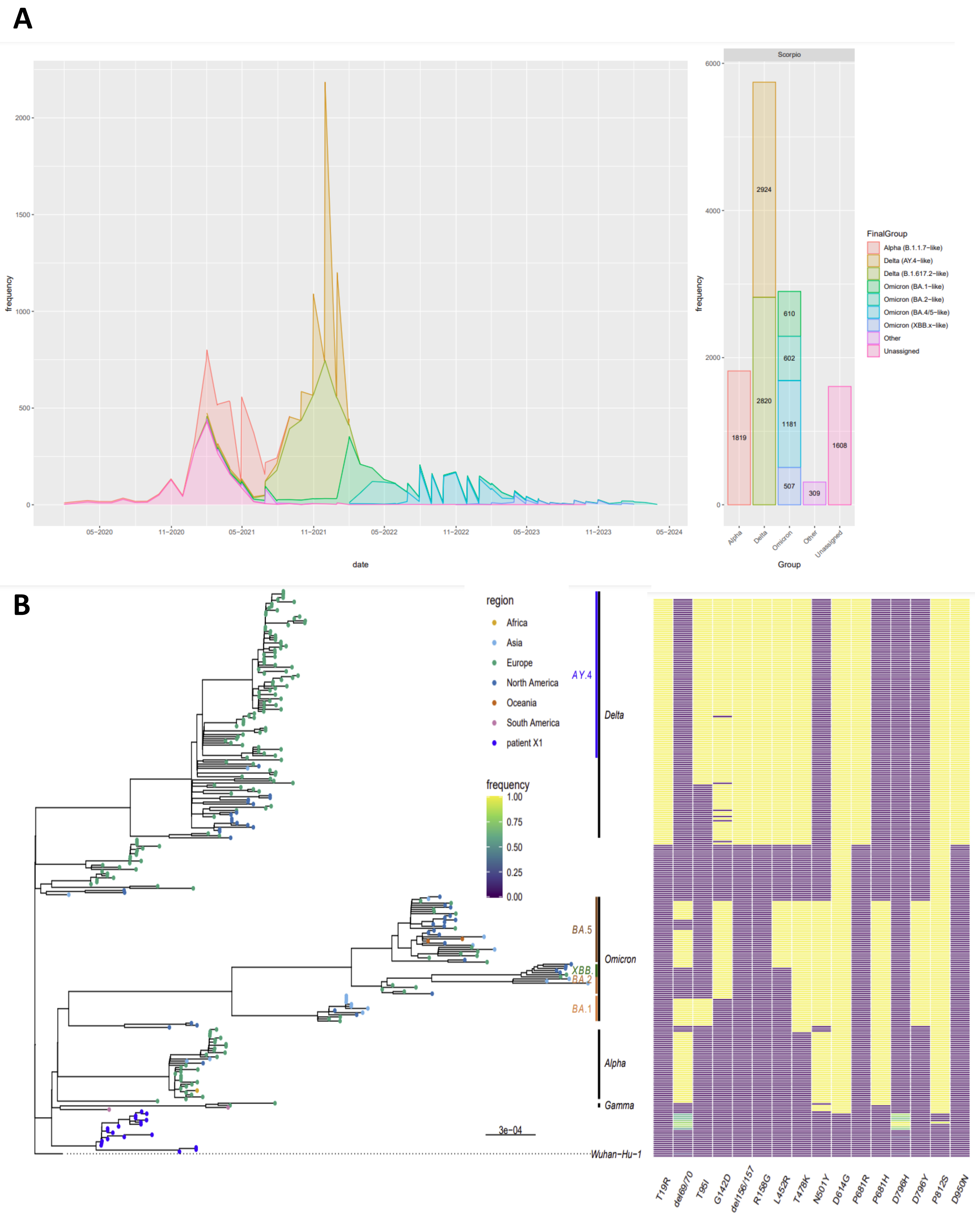

Supplementary Figure 3: Global Context of P812S containing SARS-CoV-2 global variants. A) The global prevalence of SARS-CoV-2 sequences with the S:P812S mutation in different variants defined by Scorpio as of 7th April 2024. B) Left panel - Maximum Likelihood tree (model: GTR+F+I) of patient X1 and 247 variant-stratified subsampled sequences. Tips are labelled by region. Right panel – Aligned heatmap of Spike mutations of all the subsampled sequences. For the subsampled GISAID sequences, yellow represent the presence and mauve the absence of the mutation at the consensus level.

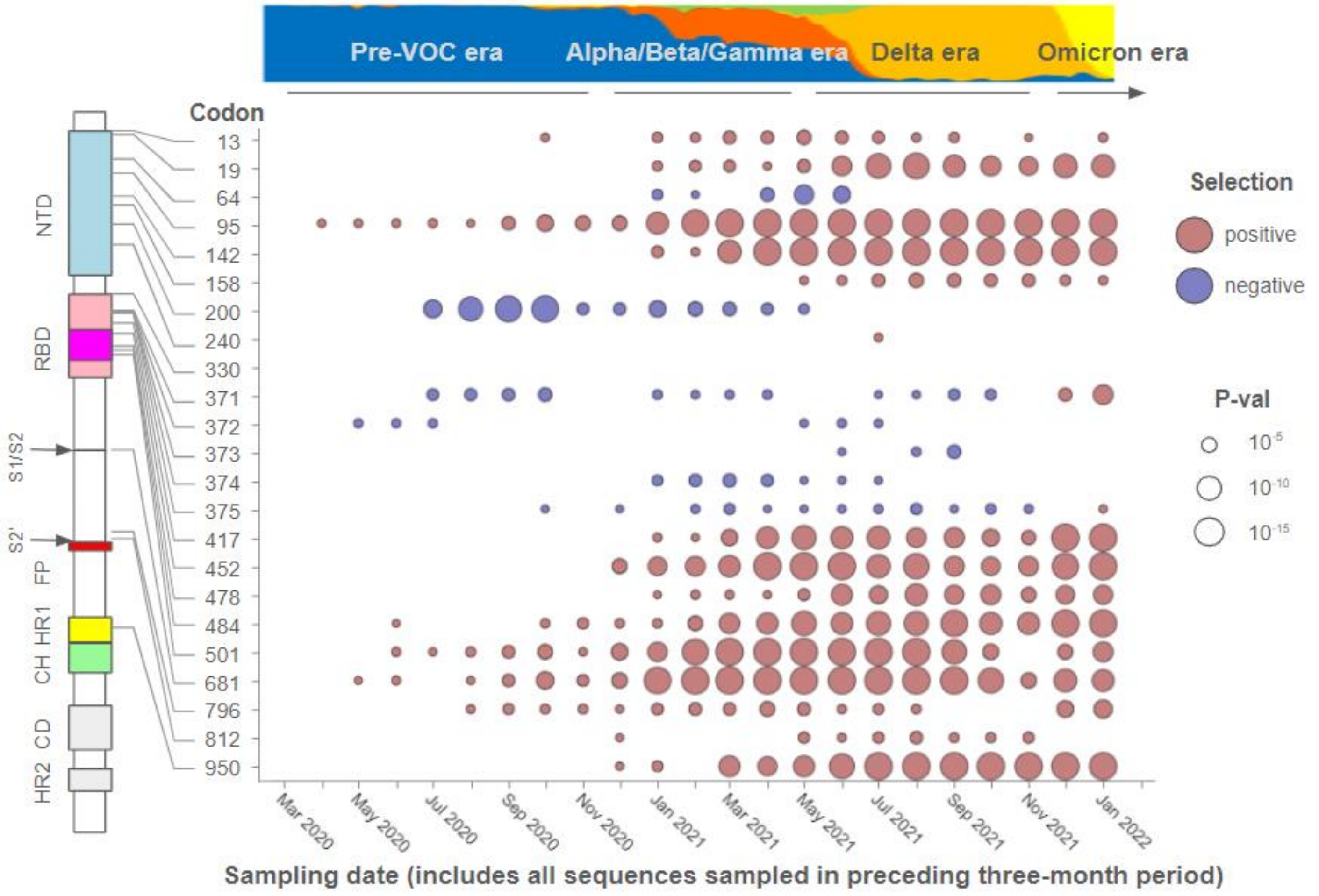

Supplementary Figure 4: Signals of natural selection at a sample of SARS-CoV-2 S-Gene codon sites during the early phases of the COVID-19 pandemic. Evidence of positive (red circles) and negative (blue circles) was detected using the IFEL method using all globally sampled near-full length SARS-CoV-2 sequences in the three months preceding the indicated sampling dates with the sizes of circles indicating the degree of statistical support for the detected selection signals (see key on the right). The approximate era during the COVID-19 pandemic (after Amodio et al) is indicated at the top. The non-variant of concern (VOC) lineages indicated in blue, the Alpha VOC in Red, the Beta VOC in dark orange, the Gamma VOC in green, the Delta VOC in orange and the Omicron VOC in yellow. The approximate location in the Spike protein of amino acids encoded by the various codon sites is indicated on the left: NTD = N-terminal domain; RBD = Receptor binding domain; FP = Fusion peptide; HR1 = Heptad repeat 1; CH = Central helix region, CD = Connector domain; HR2 = Heptad repeat 2.

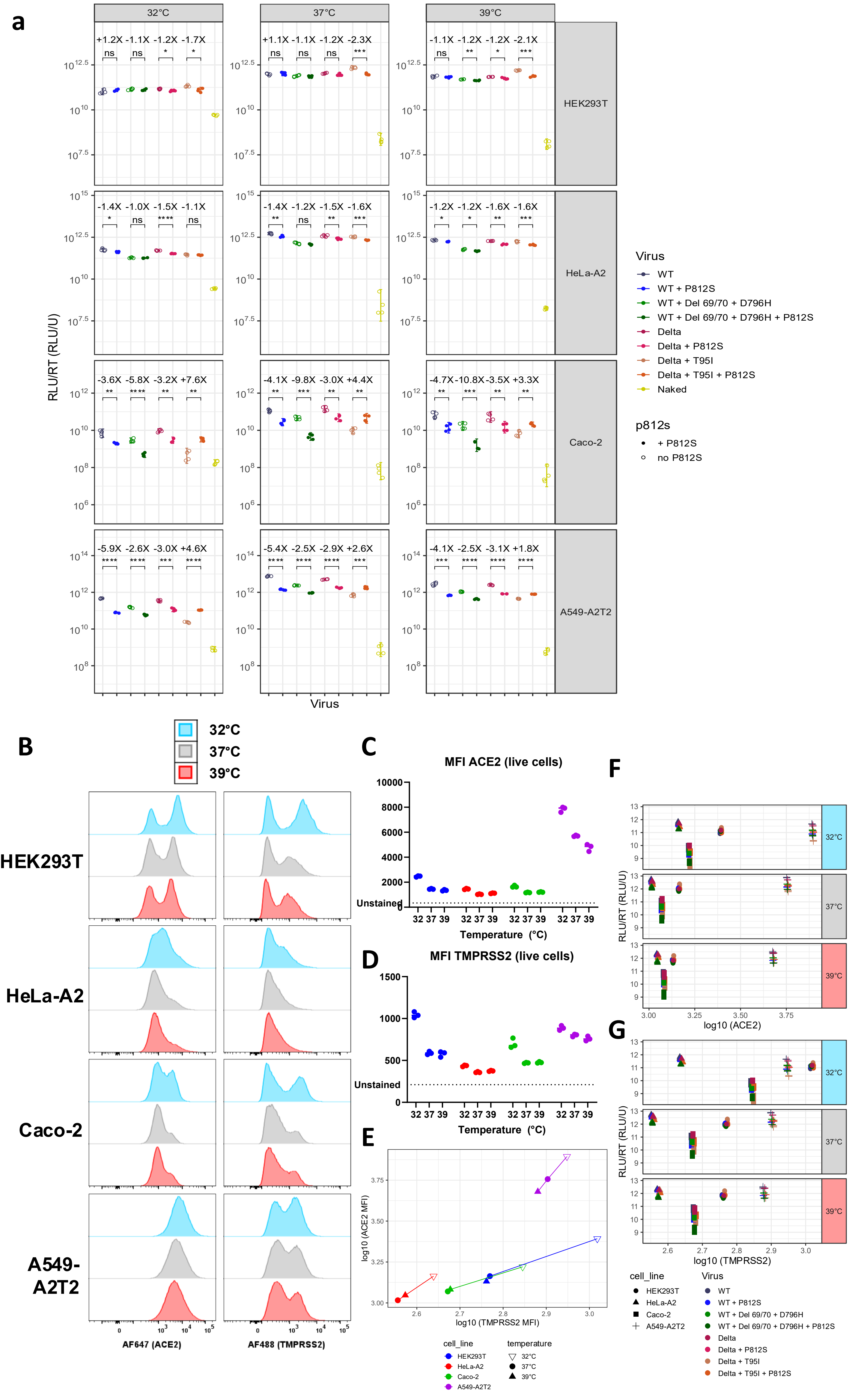

Supplementary Figure 5: P812S has a detrimental effect on cell entry in additional cell lines across 32°C, 37°C, and 39°C. A) RT-activity corrected infectivity of WT (Wuhan-Hu-1+D614G), WT+P812S, WT+Δ69/70+796H, WT+Δ69/70+796H+P812S, Delta, Delta+P812S, Delta+T95I, Delta +T95I+P812S, Naked (no spike) bearing PV introduced to cells of varying levels of ACE2 and TMPRSS levels pre-incubated at 32, 37, and 39 degrees. Data are supplemented by mean ± standard error of n = 4 replicates. Statistical analysis was performed using unpaired student's t-test with Benjamini-Hochberg correction on log-transformed values. B-E) ACE2 and TMPRSS2 surface expression of live HEK293T, HeLa-A2, CaCo-2, A549-A2T2 at 32°C, 37°C, and 39°C. b) densitogram of ACE2 and TMPRSS2 expression c,d) Mean fluorescence intensity of C) ACE2 and D) TMPRSS2 in HEK293T, HeLa-A2, CaCo-2, A549-A2T2. E) scatter plot of mean log10(ACE2) and log10(TMPRSS2) expression. F,G) RT-activity corrected infectivity of WT (Wuhan-Hu-1+D614G), WT+P812S, WT+Δ69/70+796H, WT+Δ69/70+796H+P812S, Delta, Delta+P812S, Delta+T95I, Delta +T95I+P812S plotted against log10 (ACE2) and log10 (TMPRSS2) at 32°C, 37°C, and 39°C. HEK293T, HeLa-A2, CaCo-2, A549-A2T2 are represented by shapes.

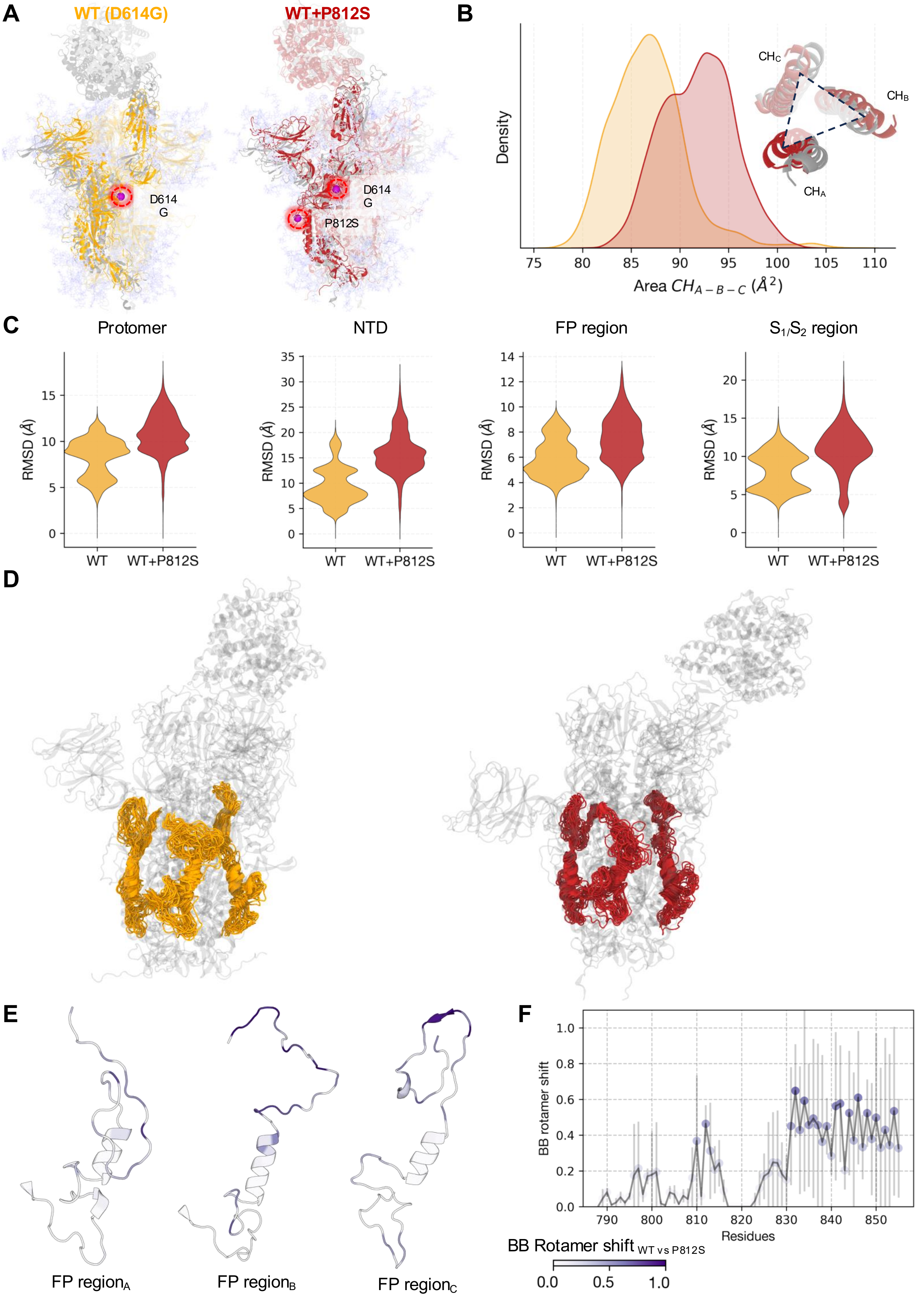

Supplementary Figure 6. The P812S mutant impacts the dynamics at both the S1/S2 and FP/S2' cleavage sites of Spike protein.
A) Snapshot of WT (D614G) and P812S spike trimers in complex with ACE-2. Both 0ns (grey) and 1μs timepoints superimposed on each other. B) The probability density plot of the triangle area formed by the centers of three CH domains is compared between WT and P812S mutant. The snapshot highlights the eagle-eye view of inter-CH orientation in WT (grey) and P812S mutant (dark red). C) The violin plots compare the distribution of RMSD calculated for the entire protomer, NTD, FP and S1/S2 regions respectively. D) The snapshots highlight the ensemble of FP conformations in WT (left) and P812S mutant (right). E) The backbone rotamer shift calculated between WT and P812S is mapped on the individual FP regions of the spike trimer. F) The corresponding rotamer shift values are plotted for FP residues. The values are averaged across three chains.

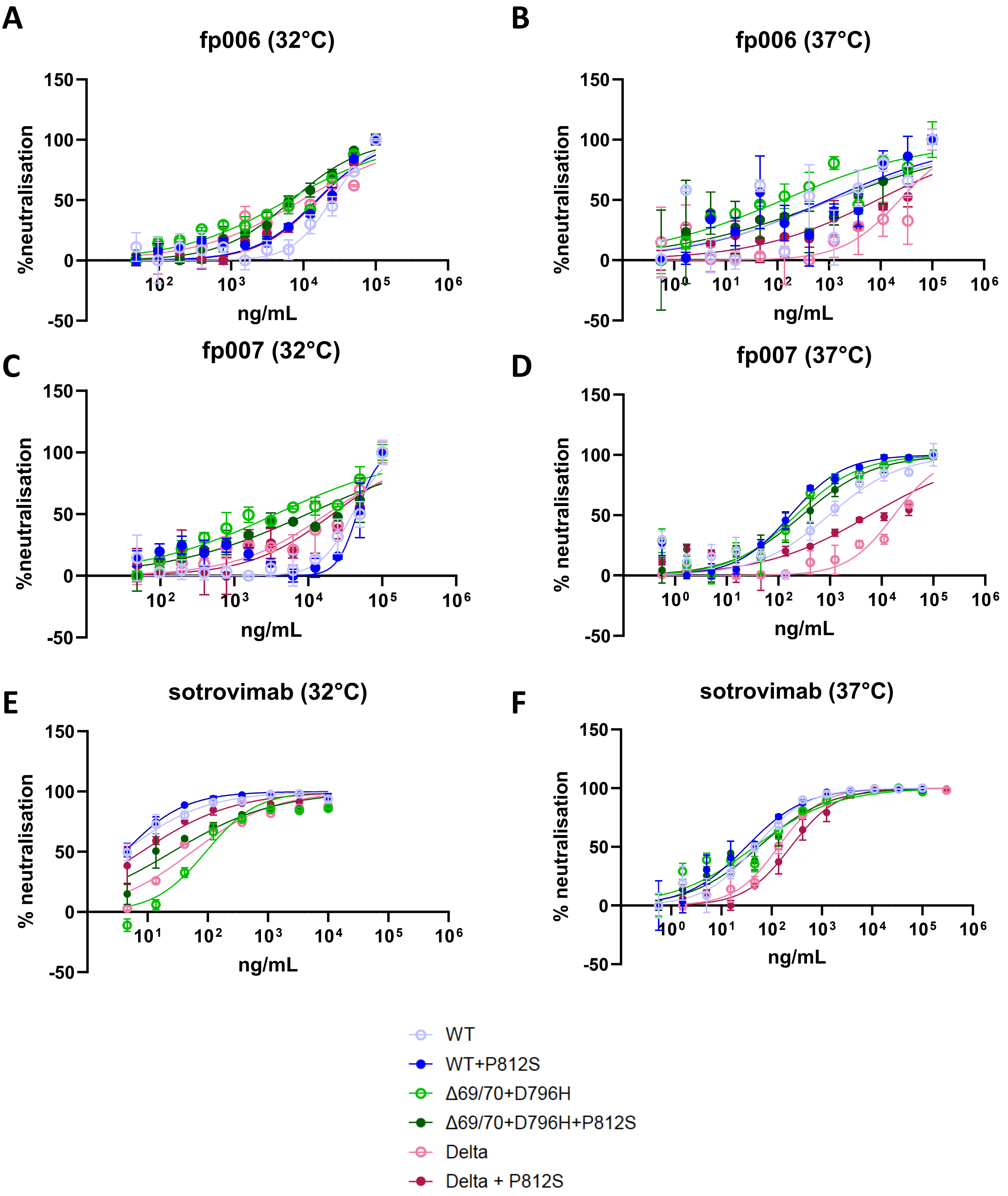

Supplementary Figure 7: Individual neutralisation curves for monoclonal antibodies fp006, fo007, and sotrovimab at 32̓C and 37°C against SARS-CoV-2 spike mutants. Results relate to Figure are representative of 2 technical replicates Data points are representative of 2 technical replicates. Error bars represent SD.

Supplementary Tables

Supplementary Table 1: Lineage of P812S-containing sequences on GISAID

scorpio_call	Aggregate Group	Number of sequences	Percentage of total P812S-containing sequences
Alpha (B.1.1.7-like)	Alpha (B.1.1.7-like)	1818	14.68%
B.1.1.7-like+E484K	Alpha (B.1.1.7-like)	1	0.01%
Delta (AY.4-like)	Delta (AY.4-like)	2903	23.45%
Delta (AY.4.2-like)	Delta (AY.4-like)	21	0.17%
Delta (B.1.617.2-like)	Delta (B.1.617.2-like)	2820	22.78%
Omicron (BA.1-like)	Omicron (BA.1-like)	610	4.93%
Omicron (BA.2-like)	Omicron (BA.2-like)	602	4.86%
Omicron (BA.5-like)	Omicron (BA.4/5-like)	1127	9.10%
Omicron (BA.4-like)	Omicron (BA.4/5-like)	54	0.44%
Omicron (XBB.1-like)	Omicron (XBB.x-like)	210	1.70%
Omicron (XBB.1.5-like)	Omicron (XBB.x-like)	162	1.31%
Omicron (XBB-like)	Omicron (XBB.x-like)	109	0.88%
Omicron (XBB.1.16-like)	Omicron (XBB.x-like)	26	0.21%
Gamma (P.1-like)	Other	167	1.35%
A.23.1-like	Other	61	0.49%
Iota (B.1.526-like)	Other	20	0.16%
Epsilon (B.1.429-like)	Other	18	0.15%
Beta (B.1.351-like)	Other	16	0.13%

Epsilon (B.1.427-like)	Other	7	0.06%
Mu (B.1.621-like)	Other	7	0.06%
Lambda (C.37-like)	Other	6	0.05%
B.1.617.1-like	Other	4	0.03%
Eta (B.1.525-like)	Other	1	0.01%
Theta (P.3-like)	Other	1	0.01%
Zeta (P.2-like)	Other	1	0.01%
Unassigned	Unassigned	1590	12.84%
Probable Omicron (Unassigned)	Unassigned	10	0.08%
Omicron (Unassigned)	Unassigned	8	0.06%
	Total	12380	

All SARS-CoV-2 isolates bearing P812S as of 7th April 2024, aggregated by Scorpio assigned lineage.

Supplementary Table 2 – half maximal effective concentration (EC50) of camostat and E64d

	**EC50 (µM)**	
**Spike presented on pseudotyped Virus**	**camostat**	**E64d**
WT	16.85	1.99
WT+P812S	23.58	3.24
Δ69/70+D796H	17.69	1.79
Δ69/70+D796H+P812S	23.76	4.50
Delta	19.45	2.11
Delta + P812S	23.59	3.87

Supplementary Table 3 – Geometric mean half-maximal neutralising titre (GMT) of AZD1222 one month post second dose vaccinee sera of P812S mutants

	**GMT**	
**Spike presented on pseudotyped Virus**	**32 °C**	**37°C**
WT	N/A	339.3
WT+P812S	N/A	347.1

Δ69/70+D796H	603.2	293.2
Δ69/70+D796H+P812S	179.8	221.7
Delta	429.8	199.9
Delta + P812S	116.1	132.3

Geometric mean half-maximal neutralising titre (GMT) of AZD1222 vaccinee sera (one month after two doses) at 32°C and 37°C against WT, WT+P812S, Δ69/70+D796H, Δ69/70+D796H+P812S, Delta, and Detla+P812S.
