# Supplementary material for: Signatures of omicron-like adaptation in early SARS-CoV-2 variants and chronic infection
Source: Cell Rep. Author manuscript; Available in PMC 2025 Dec 18. (PMC7618492; doi:10.1016/j.celrep.2025.116135)
Supplement: Supplementary Material [file EMS211251-supplement-Supplementary_Material.zip › 1-s2.0-S2211124725009064-mmc2.pdf]

Cell Reports

Article

Signatures of omicron-like adaptation in early SARS-CoV-2 variants and chronic infection

Graphical abstract

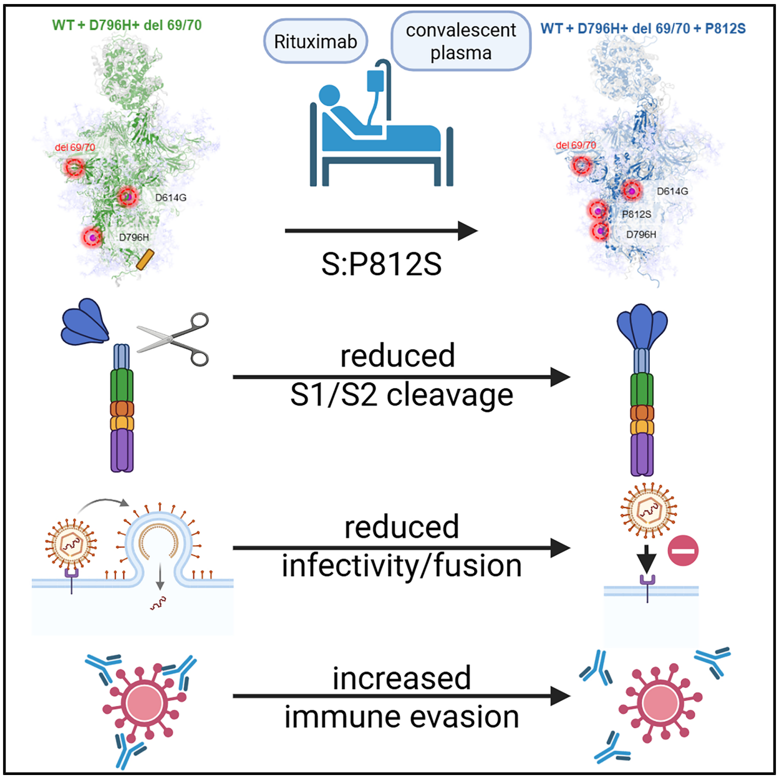

Highlights

- SARS-CoV-2 spike evolves to avoid neutralizing antibodies in chronic infection
- The mutation P812S results in escape from neutralizing antibody against fusion peptide
- The mutation P812S impairs the ability of spike to fuse cells together
- This process of immune escape with fusogenicity cost mirrors Omicron BA.1

Authors

Mark Tsz Kin Cheng, Mazharul Altaf, Jesu Castin, ..., Christopher Ruis, Lipi Thukral, Ravindra K. Gupta

Correspondence

lipi.thukral@igib.res.in (L.T.), rkg20@cam.ac.uk (R.K.G.)

In brief

Cheng et al. report SARS-CoV-2 evasion of neutralizing antibodies against the spike protein's fusion peptide region within an immune-compromised host, resulting in dramatic loss of spike-mediated fusogenicity. This mirrors the immune evasion and loss of fusogenicity of the highly successful but less pathogenic Omicron BA.1 variant.

Cheng et al., 2025, Cell Reports *44*, 116135
August 26, 2025 © 2025 The Author(s). Published by Elsevier Inc.
https://doi.org/10.1016/j.celrep.2025.116135

CellPress

Cell Reports

CellPress
OPEN ACCESS

Article

Signatures of omicron-like adaptation in early SARS-CoV-2 variants and chronic infection

Mark Tsz Kin Cheng,[1,2,12] Mazharul Altaf,[1,2,12] Jesu Castin,[3,4] Ann-Kathrin Reuschl,[5] Benjamin L. Sievers,[1,2] Kimia Kamelian,[1,2] Dejan Mesner,[5] Rebecca B. Morse,[1,2] Adam Abdullahi,[1,2] Bo Meng,[1,2] Kata Csiba,[1,2] Cambridge NIHR Bioresource, Steven A. Kemp,[1,2] Darren P. Martin,[6] Clare Jolly,[5] Christopher Ruis,[5,7,8,9] Lipi Thukral,[3,4,*] and Ravindra K. Gupta[1,2,10,11,13,*]

[1]Department of Medicine, University of Cambridge, Cambridge, UK
[2]Cambridge Institute of Therapeutic Immunology & Infectious Disease (CITIID), Cambridge, UK
[3]CSIR-Institute of Genomics and Integrative Biology, Mathura Road, New Delhi 110025, India
[4]Academy of Scientific and Innovative Research (AcSIR), Ghaziabad 201002, India
[5]Division of Infection and Immunity, University College London, London, UK
[6]Division of Computational Biology, Institute of Infectious Disease and Molecular Medicine, University of Cape Town, Cape Town 7700, South Africa
[7]Victor Philip Dahdaleh Heart & Lung Research Institute, University of Cambridge, Cambridge, UK
[8]Cambridge Centre for AI in Medicine, University of Cambridge, Cambridge, UK
[9]Department of Veterinary Medicine, University of Cambridge, Cambridge, UK
[10]Africa Health Research Institute, Durban, KZN, South Africa
[11]Hong Kong Jockey Club Global Health Institute, Hong Kong, China
[12]These authors contributed equally
[13]Lead contact
*Correspondence: lipi.thukral@igib.res.in (L.T.), rkg20@cam.ac.uk (R.K.G.)
https://doi.org/10.1016/j.celrep.2025.116135

SUMMARY

Persistent severe acute respiratory syndrome coronavirus 2 (SARS-CoV-2) infections are a source of new variants and can provide insight into evolutionary trajectories. Here, we observe upper airway-specific evolution of SARS-CoV-2, demonstrating a fusion peptide (FP) domain mutation (S:P812S) adjacent to the S2′ cleavage site that emerged during a chronic infection. Indeed, this mutation had emerged previously and been transmitted in a delta variant lineage. P812S in a spike-pseudotyped virus did not impact entry efficiency. However, cleavage at S1/S2 was reduced, and molecular dynamics simulation demonstrated altered S1/S2 loop conformations. Consistent with impaired S1/S2 cleavage, and reminiscent of Omicron BA.1, cell-cell fusogenicity was severely impaired by P812S. P812S conferred evasion of a FP-targeting monoclonal antibody, consistent with FP-region structural rearrangements. Finally, P812S-bearing viruses showed evasion of polyclonal neutralizing antibodies in sera from vaccinated individuals at 32C. These data shed light on the balance between SARS-CoV-2 upper airway adaptation/immune evasion, syncytium formation, and pathogenic potential.

INTRODUCTION

Omicron emergence represented a seismic event in the COVID-19 pandemic, demonstrating what was essentially an antigenic shift in a virus that cannot reassort its genome as influenza can. Understanding the success of Omicron is essential, and yet we have little understanding of the biological underpinnings of its ability to accommodate diverse mutations and bring together deleterious mutations to generate a highly successful new serotype. A key defining feature of Omicron in addition to immune evasion was the loss of ability of its spike to carry out cell-cell fusion.[1,2] We do not have a clear understanding of which mutations or combinations are responsible, given the multiple spike mutations observed across the protein. It is thought that this loss of syncytium formation was associated with the lower pathogenicity of Omicron and that gradual reacquisition of spike-mediated cell-cell fusogenicity by certain Omicron subvariants correlates with increased severity.[3]

In virus producer cells, full-length spike is cleaved by host furin to S1/S2 subunits, which then reassociate through electrostatic interactions. The primary receptor for severe acute respiratory syndrome coronavirus 2 (SARS-CoV-2) is ACE2, and following engagement with ACE2, the serine protease TMPRSS2 at the target cell membrane is able to cleave spike at the S2′ site, liberating the fusion peptide to initiate virus-cell fusion at the plasma membrane. The Omicron variant appears to be less dependent on TMPRSS2 and has a preference for endocytic entry, where cathepsin carries out S2′ cleavage within endosomes.[2] In addition, we have observed a cleavage defect in virus producer cells and impaired cell-cell fusion.[2] Given the broad expression of ACE2 across diverse tissues, it is unsurprising that infection of multiple organs occurs during COVID-19 infection. Local tissue

© 2025 The Author(s). Published by Elsevier Inc.
This is an open access article under the CC BY license (http://creativecommons.org/licenses/by/4.0/).

environments likely exert specific selective pressure for replication. Viral divergence in different anatomical compartments has been reported, including respiratory compartments such as the nasopharynx, sputum, trachea, and lungs,[4] and other compartments, including plasma, heart, spleen, urine, and gut,[5,6] consistent with differential selection pressure imposed by niches.[7–14] ACE2 and TMPRSS2 expression gradients exist between the upper respiratory tract (URT) and lower respiratory tract (LRT), and the efficiency of TMPRSS2 use has varied with variants of concern (VOC). Omicron showed adaptation toward the URT[2,15] and greater transmission potential. This tropism shift has been associated with reduced intrinsic disease severity.[11] Affinity toward the URT also leads to differential exposure to environmental and immune-selective pressures, creating a unique mutational spectrum that can be leveraged to determine the niche of pathogens.[5,15] Another key difference is the difference in temperature, which has an impact on spike fusogenicity and, thus, entry efficiency.[12]

SARS-CoV-2 adaptation in persistent infection of immunocompromised hosts has been demonstrated in myriad studies.[13,14,16,17] COVID-19 remains a particularly relevant threat for immunocompromised individuals, as COVID-19 vaccines have been shown to induce lower seroconversion rates,[18] reduced neutralization activity,[19] and shorter durations of protection as compared to immune-competent individuals.[20] Here, we leverage an intensively sampled case of chronic infection to report that a single mutation can have diverse phenotypic impacts and recapitulate some of Omicron's key features. P812S in the fusion peptide region and adjacent to the S2′ cleavage site has been observed to arise in a sublineage of the Delta variant and in an early B lineage yet remains uncharacterised.[21] We show that P812S causes changes in the fusion peptide (as one might expect) but also unexpected changes in the S1/S2 cleavage site that impair cleavage. Moreover, the mutation severely impairs the ability of the spike to mediate cell-cell fusion, and therefore we do not see syncytium formation. Syncytium formation is a hallmark of severe COVID-19.[22,23] Importantly and unexpectedly, the mutation also confers a degree of escape from neutralizing antibodies directed at the fusion peptide region. This is reflected in the reduced neutralization of P812S-bearing viruses by serum from vaccinated individuals, especially at the low temperatures associated with the nasopharynx. Indeed, we first isolated the mutation on an early B lineage (pre-Omicron virus) from the upper airway of a chronically infected individual but not in samples from the tracheal region, indicating that this mutation may be an adaptation to the upper airway. These properties are reminiscent of Omicron and its adaptation to upper airway transmission, with reduced severity correlating with impaired syncytium formation. Our work shows how careful genotypic and phenotypic study of chronic infections can shed light on the evolutionary biology of COVID-19.

RESULTS

Clinical course of chronic infection

We previously reported an individual treated with rituximab for a lymphoma, who had a chronic COVID-19 infection with an early Wuhan-Hu-1 + D614G SARS-CoV-2 variant (B.1.1.1) between April and August of 2020.[13] The individual was treated with three courses of remdesivir/convalescent plasma, and we previously documented immune escape conferred by D796H and viral infectivity enhancement conferred by delH69/V70 (Δ69/70). We obtained simultaneous samples from nose and throat (NT) swabs and lower endotracheal aspirates (ETAs), though previously the difference between the two sample locations were not analyzed in depth. ETA samples were only available starting from day 93 because the individual deteriorated and required intensive care admission and ventilatory support due to acute respiratory distress syndrome around day 90. From day 93 until day 101, five NT and four ETA samples were taken in total. The day 100 NT sample was removed from intra-host diversity analysis due to poor Illumina coverage and no available Ct value.

We first examined deep sequence data for the two anatomically proximate compartments of the nasopharynx and trachea. Using Shannon's entropy as a measurement of viral diversity, we observed rises in diversity in NT samples when remdesivir or convalescent plasma was administered as the infection progressed but no statistically significant difference in genetic diversity within the ETA compared to the NT samples in pooled analysis (Figures S1A–S1D). Consistent with previous studies linking Ct values to viral diversity, the increase in viral diversity was accompanied by an increase in viral load (Figure S1D).[5] On visual inspection, the intertwining sequences between NT and ETA samples meant compartmentalization was unlikely. Indeed, Slatkin-Maddison's test suggested a median of 4 migration events (95% confidence interval [CI], 2–4 migration events; $p = 1.000$ on both panmictic and structured test) on 1,000 structured permutations. This lack of compartmentalization evidence at the consensus sequence level means that it was unlikely that the diversity was a result of long-term compartmentalization but rather divergent short-term pressure in the NT and endotracheal areas on a mixing population. This is in keeping with the patient's clinical deterioration.

Nucleotide spectral analysis reveals possible mixed URT and LRT viral populations

The relative frequency of G>T mutations has been demonstrated previously to correlate with the SARS-CoV-2 replication niche, with replication in the lungs resulting in higher frequencies of G>T mutations.[15] Omicron variants, which are more URT tropic than pre-Omicron variants,[2] have lower G>T frequencies than pre-Omicron variants (Figure S2A). We therefore aimed to determine whether there was a difference in G>T frequencies between the NT and ETA isolates from the same patient. As expected given the short distance between these sites, we did not observe a difference between these niches (Figure S2B); however, we noticed a lineage of patient isolates that exhibited elevated numbers of G>T mutations (Figures 1A and 1C). This clade predominantly contains both NT and ETA sequences from late in the course of infection (after day 93), and there is a consistent increase in G>T mutations after day 64 (Figure S1C), suggesting upward spread of viruses within the patient across the respiratory tract. The elevation in G>T mutations among isolates in this lineage suggests that this spread occurred from the LRT to the URT, consistent with deteriorating lung disease. This mixing of viral populations as the patient deteriorated clinically could

CellPress
OPEN ACCESS

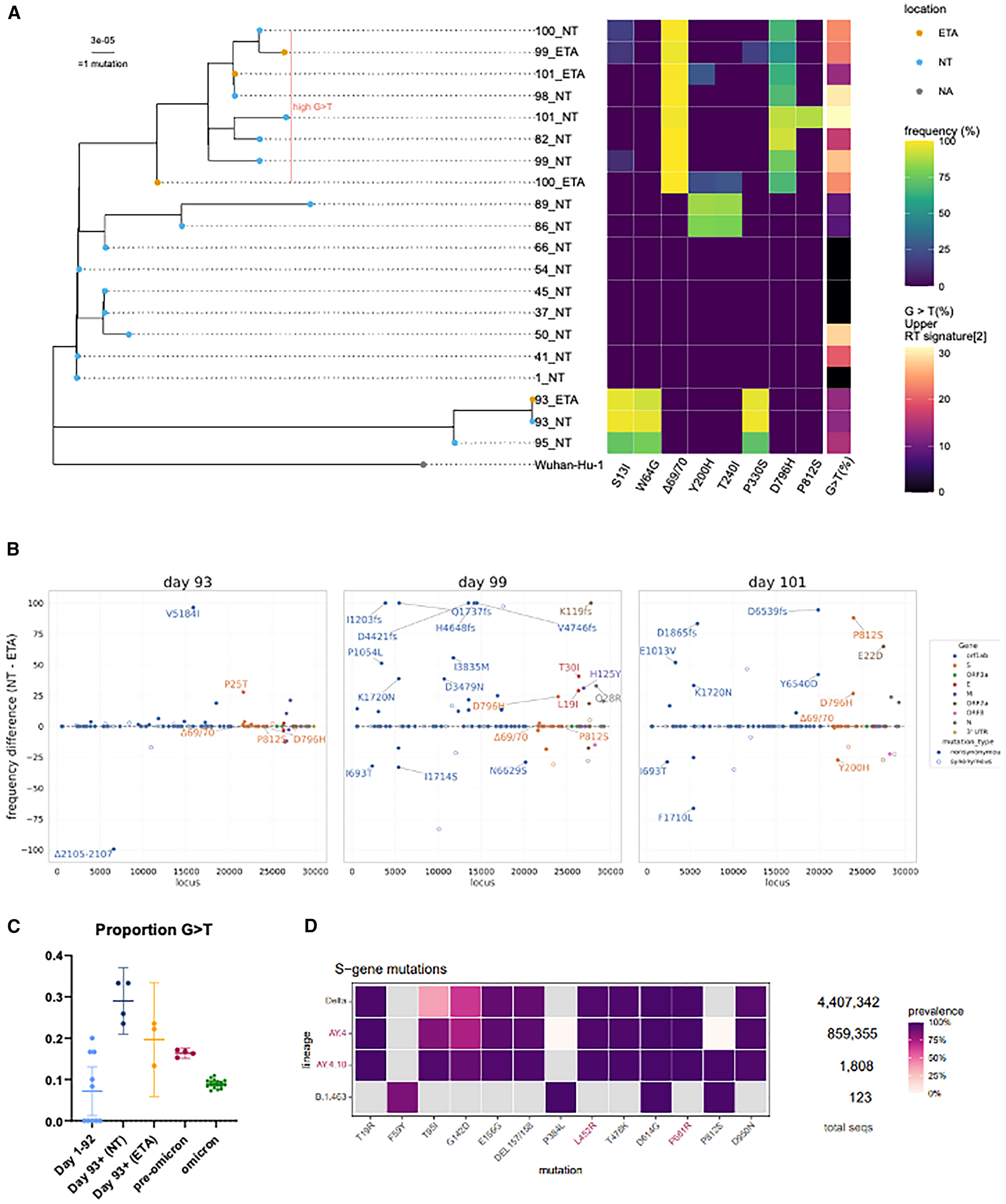

Figure 1. Genetic changes over time and across niches during chronic COVID-19

(A) Maximum-likelihood phylogenetic tree of patient X1 with the day of sampling and location (blue, NT swabs; orange, ETA) indicated. The eight-column heatmap shows the spike mutation frequency found in each sample, and the one-column heatmap shows the proportion of G-to-T mutation in each sample.

(legend continued on next page)

have been related to the physical conduit provided by the endotracheal tube or air movement associated with mechanical ventilation.

Multiple non-synonymous spike and non-spike mutations arise following therapies for COVID-19

We further examined the changes in mutation frequencies between the NT samples compared to the ETA samples. Although the long branch with S:S13I, S:W64G, and S:P330S emerged on day 93 and day 95 (Figure 1A), there is limited divergence in variant frequencies between NT and ETA samples on day 93 immediately after remdesivir administration and two units of convalescent plasma infusion (Figures 1B and S2C). On day 99, divergent mutations arose for spike (Δ69/70, D796H, and P812S) and other genes, including ORF1ab (E1013V), envelope (T19I), nucleocapsid (Q28R), and membrane (H125Y) (Figures 1B and S1E). This is likely due to selection pressure imposed by the convalescent plasma and/or remdesivir treatment.[17] Of note, the spike mutation P812S emerged on day 101 at a frequency of 88.0% in the NT sample but not in the ETA sample (Figure 1B). P812S was accompanied by a 98.1% frequency of Δ69/70 and an 89.7% frequency of 796H (Figures 1A and S2C), suggesting linkage of these three mutations on the same viral genomes.

At the time of this study (April 7, 2024), P812S had a low prevalence in the Global Initiative on Sharing All Influenza Data (GISAID) database (<0.5%, n = 12,380) (Table S1; Figure S3). P812S was most abundantly found in Delta variant viruses (n = 5744) but was also found in the Alpha variant (n = 1,819) and the major Omicron lineages BA.1 (n = 610), BA.2 (n = 602), BA.4/5 (n = 1181), and XBB (n = 507) (Table S1; Figure S3). Though P812S is a minority in these major lineages, over 99.6% of AY.4.10 sublineage sequences (1,801 of 1,808) and 99.2% of B.1.463 sublineage sequences (122 of 123) contained the P812S mutation (Figure 1D), indicating that it was a defining mutation in these lineages and arguing for possible successful transmission of P812S-bearing viruses.[24,25] Indeed, from the start of the pandemic to August 2023, spike codon 812 had an average ratio of non-synonymous to synonymous substitution rates (dN/dS ratio) of 2.47 (p = 0.184), suggesting positive selection (i.e., selection favoring changes away from the P amino acid state) (https://observablehq.com/@spond/sars-cov-2-global-genomic-selection-2019-aug-2023). Statistical evidence (p < 0.01 with the internal fixed effect likelihood, IFEL method) of positive selection at codon 812 was most marked between May and November 2021, when the Delta variant was most prevalent, corroborating the beneficial effect of diverging on a Delta genomic background from P812 to alternative amino acids such as P812S under at least some epidemiological conditions. The loss of positive selection coincided with the emergence of the Omicron variant, suggesting incompatibility with an Omicron backbone (Figure S4).

We stratified by variant, proportionally subsampled 247 SARS-CoV-2 sequences containing P812S, and inferred a maximum-likelihood tree (Figure S3B; GISAID EPI_SET_240114wm). In P812S-containing sequences, apart from the D614G mutation that arose early in the pandemic, there were no mutations consistently found to occur with P812S in the subsample. This is also evident in all sublineages with greater than 100 sequences, and P812S only defines the two lineages AY.4.10 and B.1.463 (Figure 1D).

As P812S was found only in the NT sample, we hypothesized that P812S conferred an advantage in that niche. The proximity to the S2′ cleavage site and fusion peptide, in addition to the exposed position of P812 when spike is in the open position (PDB: 6XM4; Figure 2A) prompted us to study the infectivity, cleavage, cell-cell fusion, and immune evasion properties of P812S mutants.

P812S leads to a general decrease in S1/S2 cleavage and variable cell entry efficiency

As P812S is directly upstream (N-terminal) of the S2′ cleavage site (R815/S816) and the S2 viral fusion peptide (FP; 816–855),[26–28] we hypothesized that P812S may directly impact spike glycoprotein S2 cleavage and viral entry. To test this, we introduced the P812S mutation into Wuhan-Hu-1 + D614G [wild type (WT)], WT with Δ69/70 + 796H mutations, as well as B.1.617.2 (Delta) and AY.4 (Delta + T95I). We further incorporated Delta + T95I and Delta + T95I + P812S in the panel because P812S was predominantly found in Delta (AY.4), and a previous genomic-epidemiological study showed that P812S[21] further increases the likelihood of Delta breakthrough infections.[29,30] We then performed infectivity assays using pseudoviruses with and without the P812S mutations on cell lines that varied in ACE2 and TMPRSS2 expression levels (Figures S5B–S5E). We observed little impact of P812S in cells expressing endogenous levels of receptors (Figure 2B). However, P812S was associated with a significant deficit in cell entry efficiency, on a backbone of WT, WT + Δ69/70 + 796H, Delta, and Delta + T95I, with the exception of P812S increasing infectivity in Caco-2 and A549-A2T2 cells on a Delta + T95I backbone (Figures 2B and S5A). P812S-associated entry defects persisted across 32°C, which mimics the temperature of the nasal cavity, 37°C, and 39°C, a temperature that approximates pyrexia (Figure S6).

As the cleavage of S1/S2 has been shown to correlate with infectivity/entry/transmission efficiency,[30,31] to explore this further, we performed western blot for full-length (FL) and S2 spike on HEK293T producer cell lysate and purified virions.[32]

(B) The absolute difference in percentage prevalence of mutations in patient X1 plotted against the SARS-CoV-2 genomic map. Non-synonymous mutations (filled) with >20% difference are annotated. P812S is found on day 101 in the NT compartment.

(C) Proportion of G-to-T mutations (a lower airway mutation signature) in a patient between day 1 and day 92, from day 93 onward in NT samples, from day 93 onward in ETA samples, and pre-Omicron and Omicron variants. Day 93 and day 95 were excluded, as they are separated from the main lineage. The data for Omicron and pre-Omicron variants are aggregated data from available sequences of the Ultrafast Sample placement on Existing tRees (UShER) SARS-CoV-2 phylogenetic tree. Data are plotted with mean and 95% CI.

(D) Global prevalence data on April 7, 2024, extracted via Outbreak.info enabled by data from GISAID. Spike mutations that occur at greater than 75% in any one of following lineages or sublineages, Delta (B.1.617.2), AY.4, AY.4.10, and B.1463.

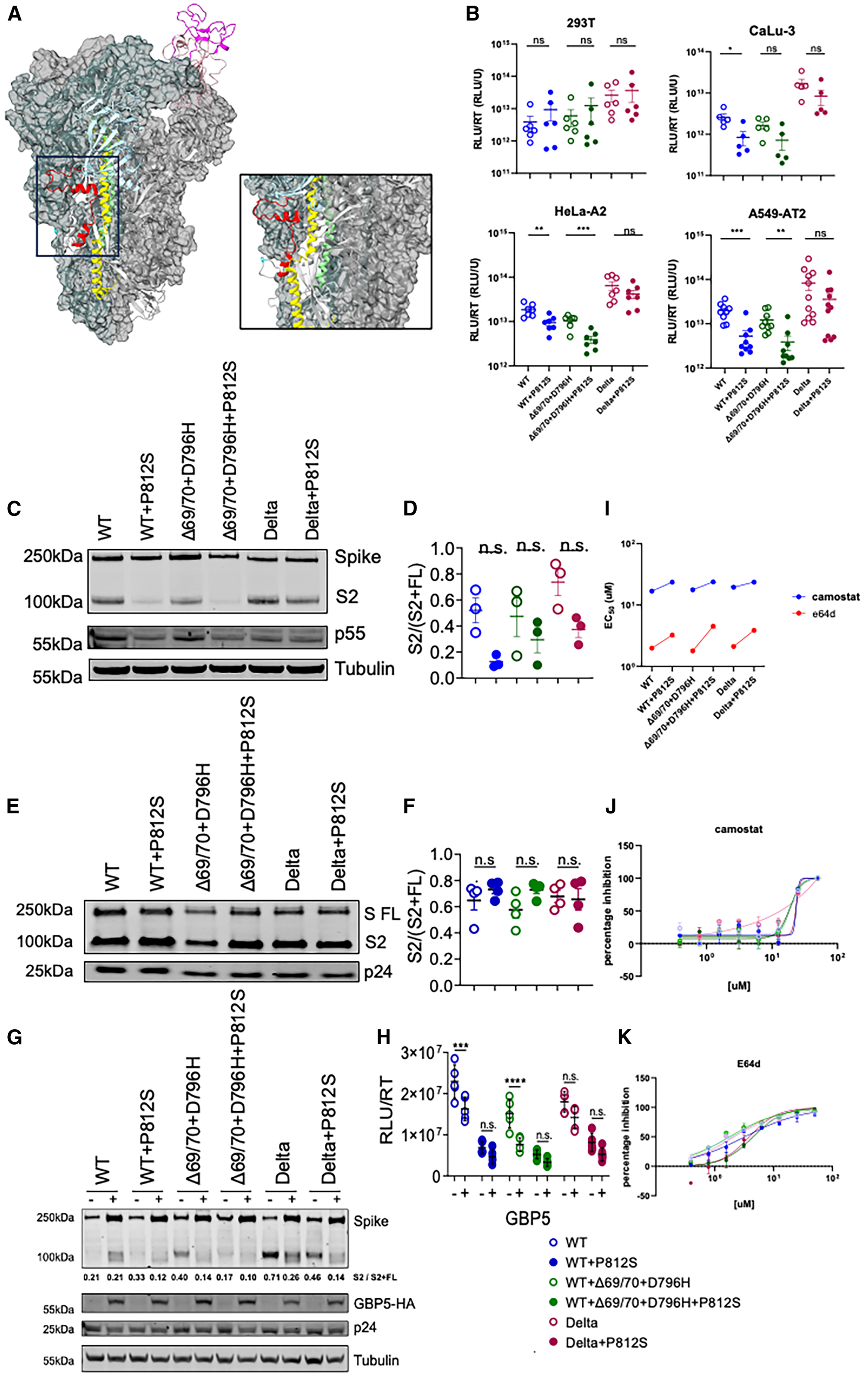

(legend on next page)

We noticed that P812S was associated with suboptimal S1/S2 cleavage in both producer cell lysate and virions, as demonstrated by a decreased ratio of S2 to total S2 + FL spike (Figure 2C). The P812S-induced reduction of S2 in cell lysate and released virions suggests that P812S may play a role in stabilizing the S protein and slowing S1 shedding, and it appears that cleaved spike is preferentially incorporated into viral particles, as we observed previously.[32] A reduction in S1/S2 cleavage is reminiscent of the change observed from the Delta to the Omicron variant,[2] which also showed a tropism shift away from cells expressing TMPRSS2 and impaired cell-cell fusion in Omicron.[15,33] We next sought to confirm whether P812S confers an entry route preference shift, as seen in the transition of global sequences from the TMPRSS2-reliant plasma membrane entry favored by the Delta variant to the cathepsin-reliant endosomal entry favored by the Omicron variant (Table S2). P812S was associated with a <2-fold resistance to both E64d (a cathepsin inhibitor, inhibiting endosomal entry) and camostat (a serine protease inhibitor, including TMPRSS2, inhibiting plasma membrane entry). In summary, we did not observe a substantial shift in efficiency of receptor or coreceptor usage for P812S.

GBP5 is an interferon-inducible protein that inhibits furin cleavage and has been shown to impact HIV envelope (Env) cleavage by furin[34,35] as well as SARS-CoV-2.[36] Given that we observed an S1/S2 cleavage defect for P812S bearing spike, we wished to explore what impact GBP5 might have on furin cleavage of the P812S spike. To test this, we co-expressed hemagglutinin (HA)-tagged GBP5 along with spikes bearing a range of mutations and packaging/reporter genome plasmids. We isolated pseudovirus (PV) and infected target A549-A2T2 lung cells lines. We observed that GBP5 reduced the infectivity of each virus tested and that the effect was smaller for PVs bearing P812S, consistent with cleavage that was already impaired at S1/2. Western blot analysis reflected these findings (Figure 2D). The successful infectivity reduction rendered by GBP5 suggests that furin cleavage still underlies the S1/S2 cleavage process of P812S mutants.

P812S severely impairs cell-cell fusion

The ability to induce syncytia via cell-cell fusion is a known property of type I virus glycoproteins, including HIV-1 Env, RSV F, and SARS-CoV-2 S proteins.[37] This process requires processing of S1/S2 in the producer cell and activity of a serine protease in the target cell to cleave S2′ and release the FP. Syncytia are thought to be a correlate of disease severity in COVID-19.[38] We have shown previously that Omicron BA.1 shifted biologically from Delta, demonstrating severely reduced cell-cell fusion activity *in vitro*, and that this appeared to correlate with S1/S2 cleavage. Subsequent Omicron variants, such as BA.4, have regained some fusogenicity.[39] Given the impact of P812S on both S1/2 cleavage and on FP structural dynamics, we measured the impact of P812S on cell-cell fusion activity using a split GFP system. As expected, Delta spike fusion was highest (Figure 3), followed by the double mutant D796H and Δ69/70 and then the WT. In accordance with the reduced infectivity and cleavage, we observed a severe defect induced by the P812S mutation on each background (Figures 2C and 2E). On the Delta backbone, T95I attenuated fusogenicity with no synergy with P812S (Figure 3).

Significant conformational rearrangements in the FP and S1/S2 cleavage site regions associated with P812S

To probe structural changes in the P812S mutant in SARS-CoV-2 spike protein, we generated two molecular dynamics simulations of the glycosylated WT and P812S spike variant based on the experimentally resolved cryoelectron microscopy structure 7A94 with 1RBD up bound to ACE2 (Figure S6). The global arrangement of the SARS-CoV-2 spike protein, which is structurally stabilized through the packing interactions of its three protomers, was significantly perturbed upon introduction of the mutation. The protomer area formed by three CH domains increased from 86.8 to 91.8 A^2 upon introduction of the P812S mutation, leading to altered spatial organization of the protomers (Figure S6B). Comparative structural analysis revealed increased dynamic fluctuations, with pronounced flexibility observed in the mutant, primarily originating from the NTD, FP, and S1-S2 regions (Figure S6C). Notably, the FP region of the SARS-CoV-2 spike protein, a short, hydrophobic segment within the S2 subunit, is positioned just downstream of the S2′ cleavage site (R815) and spans residues 816–833. To gain deeper insights into the structural changes surrounding this region, we extended our analysis to encompass residues 788–855, allowing us to capture broader conformational dynamics that may impact

Figure 2. P812S-bearing spikes maintain infectivity in cell lines expressing endogenous levels of ACE2/TMPRSS2 but show impaired S1S2 cleavage and reduced sensitivity to GBP5, E64d, and camostat

(A) Structure of SARS-CoV-2 spike (PDB: 6XM4), where two subunits are in the "RBD down" conformation (gray surfaces), and one subunit is in the "RBD up" conformation (colored ribbon). Domains are colored as follows: NTD, light blue; RBD, pink; RBM, magenta; P812, cyan; FP, red; HR1, yellow; HR2, green.

(B) PV entry of WT (Wuhan-Hu-1 + D614G), WT + P812S, WT + Δ69/70 + 796H, WT + Δ69/70 + 796H + P812S, Delta, and Delta + P812S introduced into HEK293T, Hela-A2, A549-AT2, and Calu-3 cells at 37°C. Data are supplemented by mean ± standard error of 3–8 technical replicates. Statistical analysis was performed using unpaired Student's t test.

(C–F) Western blot analysis of spike cleavage in producer HEK293Tv cell lysate (C) and isolated PV (E). S2-to-FL spike ratio was analyzed by densitometry (ImageJ), and S2/S2 + FL cleavage proportion was calculated for virions (D) and cell lysates (F).

(G and H) GBP sensitivity of spike-Gag-PVs (produced in the presence or absence of GBP5 on ACE2/TMPRSS2-A549. Western blot of transfected producer cell lysates was probed with antibodies to S2, HA, p24, and tubulin (G). PV infectivity (relative luciferase units [RLU]/unit of reverse transriptase activity - RLU/RT measured in U) is representative of two independent experiments with 5–6 measurements each (H). Statistical analysis was performed using one-way ANOVA with Bonferroni correction.

(I–K) Half-maximal effective concentration (EC50) of camostat (TMPRSS2 inhibitor) and E64d (cathepsin inhibitor). individual EC50s of Camostat (blue) and E64d (red) in A549-ACE2/TMPRSS2, against PV expressing respective Spike (I). individual EC50 curves against each virus of camostat (J) and E64d (K). Data are representative of two independent experiments.

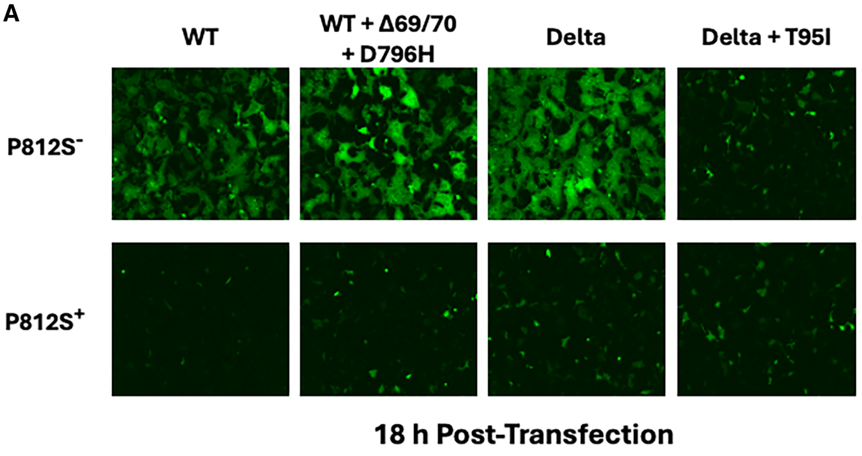

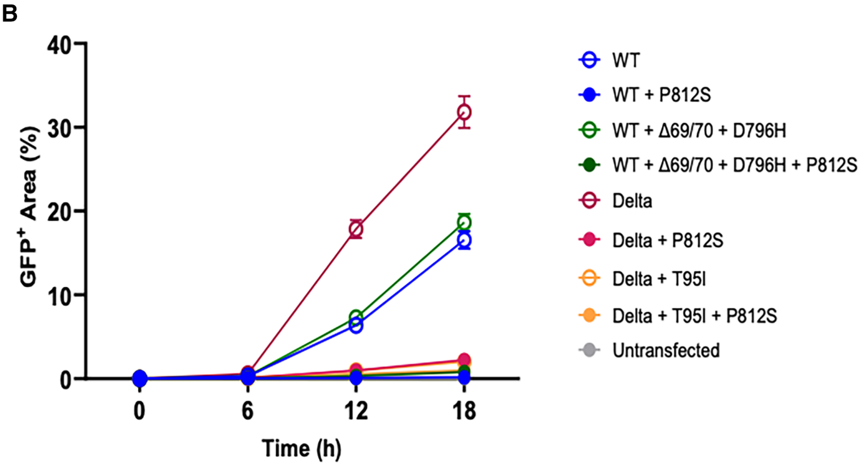

Figure 3. P812S displayed impaired cell-cell fusion activity

(A) Representative images of spike-mediated cell-cell fusion in WT (Wuhan-Hu-1 + D614G), WT + P812S, WT + Δ69/70 + 796H, WT + Δ69/70 + 796H + P812S, Delta, Delta + P812S, Delta + T95I, and Delta + T95I + P812S 18 h post transfection. Images were captured using the IncuCyte SX5 Live-Cell Analysis System (Sartorius).

(B) Quantification of spike-mediated cell-cell fusion, showing the percentage of GFP$^+$ area to the total cell area 0, 6, 12, and 18 h post transfection. Cell-cell fusion was determined as the proportion of green area to the total phase area and was calculated with the IncuCyte analysis software (Sartorius). Data are mean ± SEM from four fields of view at each time point and are representative of two independent experiments.

fusion efficiency and viral entry. Furthermore, the region of S1/S2 proteolytic cleavage site (residues 669–696) also showed greater structural compaction in the P812S mutant compared to the WT, suggesting potential alterations in cleavage accessibility and spike activation dynamics. To understand the residues that are responsible for the local perturbation at the FP region (Figure S6), we monitored the backbone conformational changes and computed rotamer shift with respect to the WT (explained in STAR Methods). The shift is majorly elevated in the loop flanking the FP helix, which is comprised of residues 830–855, as shown in Figures S6E and S6F. These structural modeling studies provide insight into our observations of impaired S1/S2 cleavage, consistent with our understanding that proteolytic cleavage at the S2′ site may be impacted by the P812S mutation.

P812S confers evasion of serum neutralizing antibodies

Multiple broadly neutralizing antibodies have been identified to interact with amino acids on the N-terminal side of the FP (816–655), including P812.[40–42] In addition, P812 has been identified as a highly exposed epitope.[43] Therefore, we hypothesized that P812S may confer a degree of immune escape due to selective pressure imposed by convalescent plasma treatment. We tested the neutralization sensitivity of Wuhan-Hu-1 + D614G (WT), WT + P812S, WT + Δ69/70 + D796H, and WT + Δ69/70 + D796H + P812S, using a previously reported PV system.[44] We tested 20 stored vaccinee sera (average age, 66.0; 55% M) 1 month after the second-dose AstraZeneca ChAdOx1 COVID-19 vaccine.[45,46]

We confirmed previous observations[13] showing modest immune escape by the double mutant Δ69/70 + D796H, with a 1.2-fold decrease in geometric mean serum neutralizing titers between WT and WT + Δ69/70 + D796H (Figure 5A). P812S on a WT + Δ69/70 + D796H backbone was associated with a further loss of sensitivity to neutralizing serum antibodies (Figure 5A). This suggests that the immune evasion capabilities conferred by D796H are enhanced by P812S.

To better understand the impact of the P812S mutation, we produced Delta pseudovirions with and without the P812S mutation. Using sera 1 month after two doses of AstraZeneca ChAdOx1-S, both baseline serum neutralizing titers against Delta and Delta + P812S were lower than in the WT (Table S3). There was a significant decrease in serum neutralization GMT of the Delta P812S mutant compared to Delta, consistent with immune evasion (Figure 5A). In summary, at 37°C, in both the Delta and WT + Δ69/70 + D796H backbones, P812S incorporation conferred a degree of resistance to serum neutralization. To better simulate the upper airway environment, we tested PVs expressing WT + Δ69/70 + D796H or Delta ± P812S against serum/monoclonal antibody at 32°C.[47] At 32°C, P812S conferred some resistance to vaccinee serum by ×1.4-fold in the background of WT + Δ69/70 + D796H and ×1.3-fold in the Delta background, respectively (Figure 5B; Table S3).

Finally, we wished to explore the impact of P812S on susceptibility to sotrovimab treatment, given that it was the only licensed therapeutic monoclonal during chronic infection.

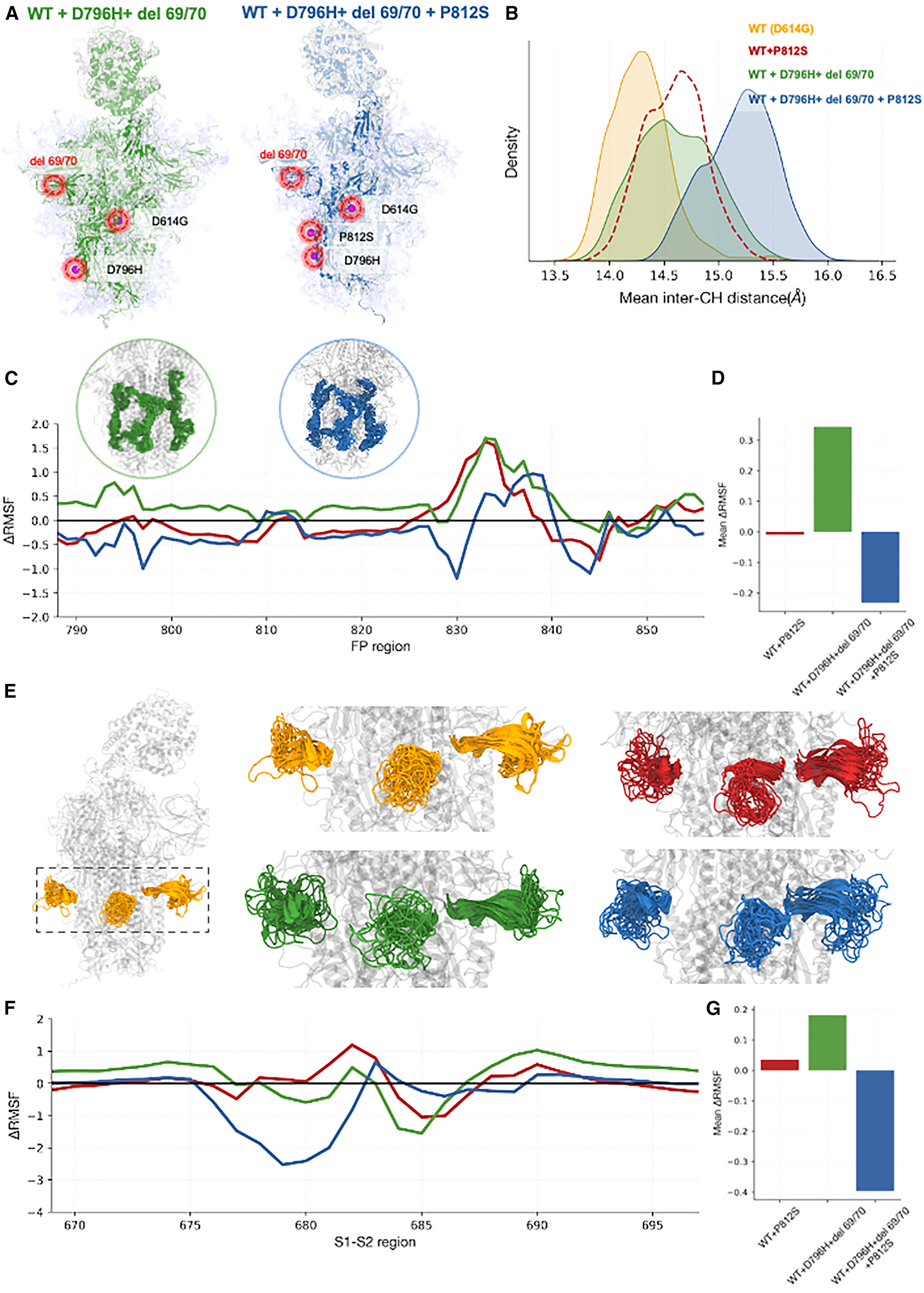

(legend on next page)

Sotrovimab (derived from S-309) (GlaxoSmithKline, GSK) is a monoclonal class 3 antibody that targets a highly conserved epitope within the spike receptor binding domain (RBD).[48] In agreement with previous literature, sotrovimab demonstrated similar neutralization titers with serum against the WT and Delta,[49] and P812S had a limited effect on sotrovimab susceptibility, with IC50 differences of less than 2-fold in the presence versus absence of P812S (Figures 5C–5F and S7C–S7F).

P812S confers evasion of neutralizing antibodies targeting the FP

To gain some understanding of the mechanism of P812S in immune evasion, two monoclonal antibodies were tested. We used the FP-targeting recombinant monoclonal antibodies fp.006 and fp.007 from Bianchini et al.[28] In brief, fp.006 recognizes a conserved FP epitope that is only exposed upon ACE2 binding. IC50 reduction conferred by P812S for the WT (×1.22) and Delta (×2.14) is modest for the ACE2-binding dependent fp.006 compared to the IC50 increase for Δ69/70 + D796H (×8.88). This suggests that, on the background of Δ69/70 + D796H, P812S decreased sensitivity of the FP epitope to neutralizing antibodies in serum but not on the Wuhan-Hu-1 + D614G (WT) background.

Antibody fp.007 binding affinity and neutralizing capacity are independent of ACE2 binding. fp.007 demonstrated a 5.58- and 2.96-fold decrease in IC50 upon P812S introduction on both WT and Delta backbones, respectively, with little difference upon P812S introduction to the Δ69/70 + D796H double mutant (Figures 5C–5E; Table S3). Taken together it appears that FP antibodies are likely to have driven selection of P812S, especially as P812S induced significant conformational changes in the FP region.[50] In order to probe this further, we performed molecular dynamics (MD) simulations of the Δ69/70 + D796H double mutant with and without P812S (Figure 4A). The average interprotomer distance increased to 15.2 Å in the presence of P812S along with background mutations (Figure 4B). Although the perturbations were not as high as observed in P812S without background mutations (Figure S6), we captured elevated fluctuations at the FP and S1/S2 regions. We computed ΔRMSF ($RMSF_{WT}$-$RMSF_{MUT}$) to assess the changes in the fluctuation patterns upon mutation. In the FP region, P812S, along with background mutations, introduces local instability with an up to 1 Å increase in RMSF (Figures 4C and 4D). We could capture relatively increased fluctuations in the S1/S2 region (up to ~3 Å) in P812S + background mutations. While the presence of P812S imparted instability to FP and S1/S2 sites, the lack of P812S (Δ69/70 + D796H double mutant) showed no major changes in the fluctuations (Figures 4E–4G). These observations support the observed escape from the FP-targeting antibody and the reduced S1/S2 cleavage seen for the triple mutant.

At 32°C, the evasion of fp006 and fp007 conferred by P812S is smaller for Δ69/70 + D796H, supporting the emergence of Δ69/70 + D796H + P812S from a population mixture rather from compartmentalization. On the other hand, at 32°C, P812S grants increased resistance to Delta against both FP-specific fp006 and fp007 at the cost of increased sensitivity to the RBD-specific sotrovimab, suggesting a potential mechanism of fitness gained in Delta and Delta sublineages, including AY.4 (Figures 5D and 5F).

DISCUSSION

Persistent SARS-CoV-2 infections are a likely source of new variants and may provide valuable insight into past and future evolutionary trajectories, in particular those involving allosteric interactions that defy genotype-to-phenotype prediction. Here, we observe upper airway-specific evolution of SARS-CoV-2, demonstrating FP domain mutation S:P812S adjacent to the S2′ cleavage site that emerged during a chronic infection in an immunocompromised individual. This mutation had also emerged previously in an ancestral B lineage as well as the Delta variant lineage and has been transmitted successfully. P812S in spike pseudotyped virus particles decreased entry efficiency across cell lines expressing a range of ACE2 and TMPRSS2. This is likely due to the efficiency of spike cleavage at S1/S2 being reduced, and molecular dynamics simulation demonstrated an altered S1/S2 loop conformations that likely impacted furin-mediated cleavage. We noted that GBP5, an interferon-inducible restriction factor that inhibits furin-mediated cleavage, had a smaller impact on inhibition of P812S-bearing pseudotyped viruses, consistent with a pre-existing S1/S2 cleavage defect in P812S-bearing spikes.

Consistent with this observation and reminiscent of Omicron BA.1, cell-cell fusogenicity was severely impaired by introduction of P812S. The mutation also introduced significant perturbations to the FP region and protomer-protomer packing. On the other hand, P812S conferred evasion of a neutralizing monoclonal antibody targeting the FP, consistent with significant structural rearrangements in the FP region. As the S2 subunit and FP region are strong candidates for pan-sarbecoviruses broadly neutralizing antibody therapy and vaccines,[28,40,41,51] our work shines light on potential functional deficits associated with immune escape mutations.

Finally, P812S-bearing viruses showed evasion of polyclonal neutralizing antibodies in serum from vaccinated individuals at 32°C (simulating the URT) and, to a lesser extent, at 37°C. We propose that the selection of P812S in the upper airway compartment is an immune evasion adaptation that came at

Figure 4. Presence of P812S imparts instability to the S1/S2 cleavage and FP regions
(A) The snapshots highlight the background double mutant (D796H + del 69/70) with (right) and without (left) P812S. The position of mutations is highlighted.
(B) The average inter-CH distance distributions are plotted for the WT and all three mutants.
(C) ΔRMSF ($RMSF_{WT}$-$RMSF_{MUT}$) is plotted for residues of the FP region. The snapshots highlight the conformational ensemble of the FP region seen in the background mutations with and without P812S substitution.
(D) The average ΔRMSF from the FP region is plotted for all mutants.
(E) The snapshots highlight the dynamics of the S1/S2 region in the WT and three mutations.
(F) ΔRMSF is plotted for the S1/S2 region, highlighting all three mutants.
(G) The average ΔRMSF underlying S1/S2 residues is plotted for all mutants.

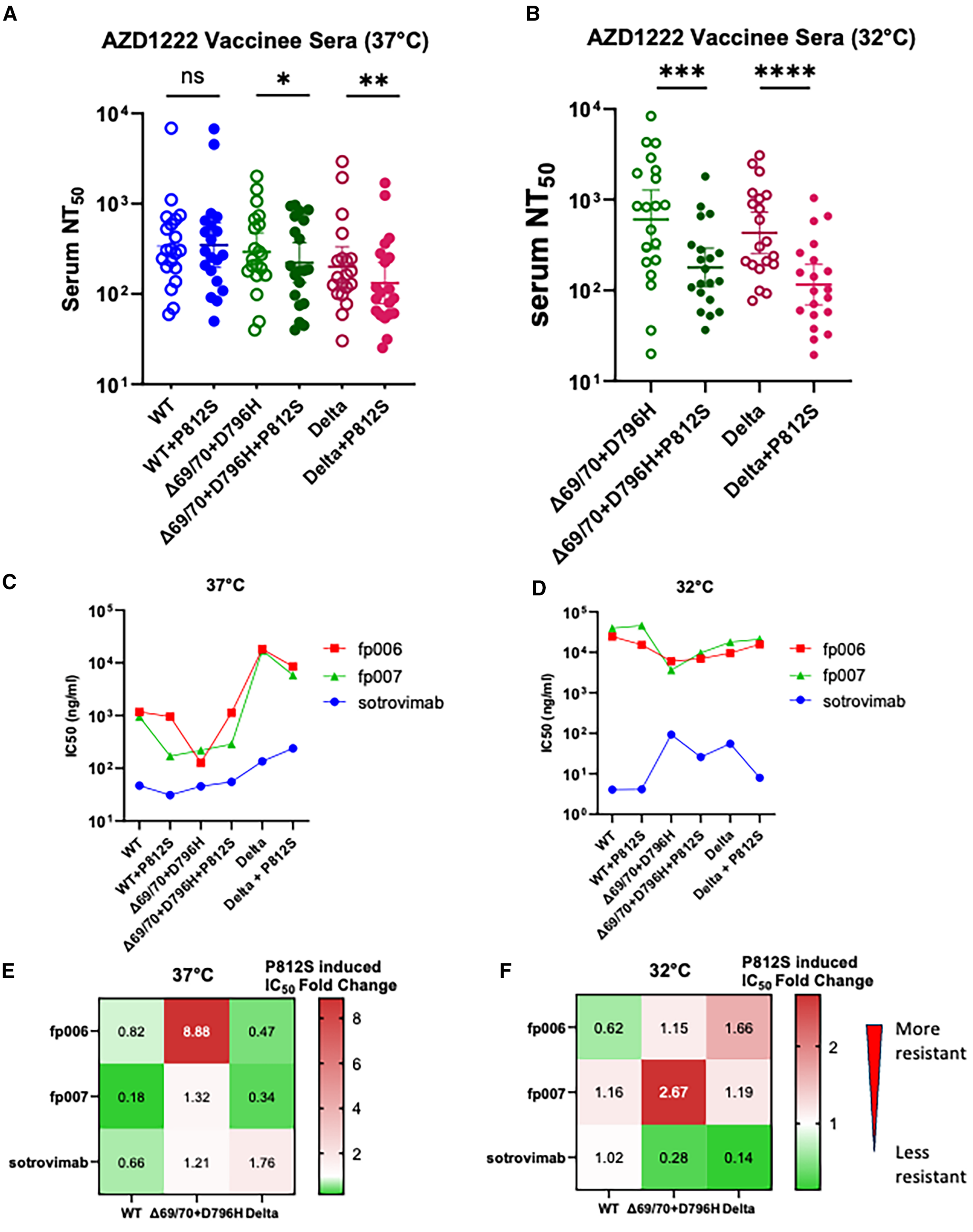

Figure 5. P812S confers escape from polyclonal sera from vaccinated individuals and neutralizing antibodies against the FP

(A and B) Neutralization by second-dose adenoviral vector (AZD1222) vaccinee-elicited sera against the WT (Wuhan-Hu-1+D614G), WT + P812S, WT + Δ69/70 + 796H, WT + Δ69/70 + 796H + P812S, Delta, and Delta + P812S pseudotyped virus. The PV was co-incubated with serum for 1 h at 37°C (A) or 32°C (B). Reciprocal geometric mean titer is shown with 95% CI. Statistical analysis was performed using ratio paired t test corrected for multiple comparisons using the Holm-Šídák method. $*p < 0.05$, $**p < 0.001$, $***p < 0.0001$.

(C and D) IC50 of fp006 (red), fp007 (green), and sotrovimab (blue) incubated with PVs corresponding to the indicated mutations at 37°C (C) or 32°C (D). Results are representative of 2 technical replicates.

(E and F) The IC50 fold change induced by adding P812S to each spike backbone are represented in the heatmap at 37°C (E) or 32°C (F).

the cost of cell-cell fusion and entry efficiency in some cells overexpressing ACE2/TMPRSS2, possibly mediated through impaired S1/S2 cleavage in P812S-bearing spikes.

Interestingly, this URT adaptation occurred on the background of within-host evolution, suggestive of adaptation to the LRT based on mutational spectrum analysis. Thus, it is plausible that the LRT population migrated to the URT, given the intense inflammation in the lower airway in the present case. Although we were unable to separate URT and LRT isolates in mutational spectra due to the similarity of sequences at the consensus level from our patient, comparison of patient sequences and global sequences of pre-Omicron and Omicron lineages is suggestive of progression from the lower to the upper airway. Our work demonstrates the importance of studying compartmentalization of viral populations, especially in persistent infection. Monitoring mutations that have epistatic effects could potentially assist in prediction of mutations in future circulating strains. The driving force for mutations in the immunocompromised host is significantly different and allows for unique combinations, especially given suboptimal antibody-driven selection pressure. We speculate that, with high levels of viral replication and genetic diversification, P812S was selected for its immune-evasive properties and that the accompanying reduced entry efficiency was counterbalanced by increased infectivity at pyrexial temperatures (Figure S5A) during disease progression.

It is notable that a single mutation can have multiple unexpected phenotypic impacts. These observations provide clues as to the events leading to the emergence of the highly successful Omicron variant, highlighting the role of allosteric impacts of immune escape mutations (for example, on cleavage sites) and the importance of mutational context. Indeed, the dogma that viruses become less pathogenic over time is widely quoted, and yet the evidence for this is weak. If syncytium formation indeed is a correlate of disease severity, then our data provide one theory for how such a process might occur for SARS-CoV-2, although, ultimately, the "holy grail" is a step-by-step understanding of how the Omicron variant selected individual mutations.

Limitations of the study

Our study was based on lentiviruses pseudotyped with different SARS-CoV-2 spikes. Although consistency has been observed between the pseudotyped virus system and the live-virus system for the study of virus entry,[45,52] there may still be potential effectors only present in the live-virus system.[2] Confirmation of our finding in a live-virus system would be desirable.

RESOURCE AVAILABILITY

Lead contact

Requests for further information should be directed to and will be fulfilled by the lead contact, Ravindra K. Gupta (rkg20@cam.ac.uk).

Materials availability

This study did not generate new unique reagents.

Data and code availability

Raw sequencing data have been reported previously by Kemp et al.[13] This paper does not report original code or software. All computational methods used have been referenced and are publicly available. Any additional information to reanalyze the data reported is available from the lead contact upon reasonable request.

CONSORTIA

The members of CITIID-NIHR BioResource COVID-19 Collaboration are John R. Bradley, Stephen Baker, Barbara Graves, Hannah Stark, Sabine Hein, Ingrid Scholtes, Daniela Caputo, Anne Elmer, Emma Le Gresley, Nathalie Kingston, Patrick Chinnery, Daniel Cooper, Gordon Dougan, Ian Goodfellow, Nathalie Kingston, Paul J. Lehner, Paul A. Lyons, Nicholas J. Matheson, Caroline Saunders, Kenneth G. C. Smith, Charlotte Summers, James Thaventhiran, M. Estee Torok, Mark R. Toshner, Michael P. Weekes, Gisele Alvio, Sharon Baker, Areti Bermperi, Karen Brookes, Isabel Cruz, Ranalie de Jesus, Katie Dempsey, Giovanni Di Stephano, Jason Domingo, Sarah Hewitt, Heather Jones, Sherly Jose, Jenny Kourampa, Caroline McMahon, Vivien Mendoza, Charmain Ocaya, Ciro Pasquale, Marlyn Perales, Carla Ribeiro, Vargas, Bensi Vergese, Laura Watson, Jieniean Worsley, Julie-Ann Zerrudo, Laura Bergamashi, Gräf, Kelvin Hunter, Federica Mescia, John Allison, Heather Biggs, Helen Butcher, Matt Chandler, Debbie Clapham-Riley, Eleanor Dewhurst, Christian Fernandez, Anita Furlong, Jennifer Gray, Tasmin Ivers, Rachel Linger, Mary Kasanicki, Rebecca King, Sarah Meloy, Alexei Moulton, Francesca Muldoon, Nigel Ovington, Sofia Papadia, Christopher J. Penkett, Isabel Phelan, Venkatesh Ranganath, Roxana Paraschiv, Abigail Sage, Jennifer Sambrook, Katherine Schon, Kathleen E. Stirrups, Paul Townsend, Neil Walker, Jennifer Webster, Petra Polgarova, Sarah L. Caddy, Laura G. Caller, Yasmin Chaudhry, Martin D. Curran, Theresa Feltwell, Stewart Fuller, Iliana Georgana, Grant Hall, William L. Hamilton, Myra Hosmillo, Charlotte J. Houldcroft, Rhys Izuagbe, Aminu S. Jahun, Fahad A. Khokhar, Anna G. Kovalenko, Luke W. Meredith, Surendra Parmar, Malte L. Pinckert, Anna Yakovleva, Emily C. Horner, Lucy Booth, Alexander Ferreira, Rebecca Boston, Robert Hughes, Juan Carlos Yam Puc, Nonantzin Beristain-Covarrubias, Maria Rust, Thevinya Gurugama, Lihinya Gurugama, Thomas Mulroney, Sarah Spencer, Zhaleh Hosseini, and Kate Williamson.

ACKNOWLEDGMENTS

This work was funded by a Wellcome Senior Fellowship (WT108082AIA to R.K.G.). This research was supported by the NIHR Cambridge Biomedical Research Centre (NIHR203312). A.-K.R. and C.J. are funded by a Wellcome Investigator award (223065 to C.J.). L.T. is thankful for funding support from the DBT/Wellcome Trust India Alliance (IA/21/2/505925). L.T. is also thankful to the funding support from the DBT NNP project (BT/PR40265/BTIS/137/62/2023) and CSIR-IGIB institutional support from OLP2503. The views expressed are those of the authors and not necessarily those of the NIHR or the Department of Health and Social Care. The research was supported by the Hong Kong Jockey Club Global Health Institute (HKJCGHI), Hong Kong Special Administrative Region, China. We thank Dr. Maximillian Muenchhoff for valuable discussions on SARS-CoV-2 HLA and CD8 T cell epitope evasion analysis. We also thank Dr. Filippo Bianchini, Ms. Jasmine Chantal Cantergiani, and Dr. Davide F. Robianni for sending samples and expression plasmids of fp.006 and fp.007 antibodies.

AUTHOR CONTRIBUTIONS

Conceptualization, M.T.K.C. and R.K.G.; methodology, M.T.K.C., D.P.M., C. R., B.M., M.A., and J.C.; investigation, M.T.K.C., M.A., D.P.M., C.R., B.L.S., K.K., R.B.M., M.A., K.C., J.C., and A-K.R.; writing – original draft, M.T.K.C. and R.K.G.; writing – review & editing, M.T.K.C., M.A., D.P.M., C.R., L.T., R.

K.G., A.-K.R., and C.J.; funding acquisition, R.K.G. and L.T.; resources, R.K.G. and L.T.; supervision, B.M., S.A.K., R.K.G., C.J., and J.C.

DECLARATION OF INTERESTS

The authors declare no competing interests.

STAR★METHODS

Detailed methods are provided in the online version of this paper and include the following:

- KEY RESOURCES TABLE
- EXPERIMENTAL MODEL AND STUDY PARTICIPANT DETAILS
  - Ethical approval
  - Cell culture
  - Clinical sample collection and sequencing
- METHOD DETAILS
  - Sequence data processing and analysis
  - Genetic diversity and viral load analysis
  - Phylogenetic analysis and lineage assignment
  - Signals of natural selection in the SARS-CoV-2 S-gene
  - Structure analysis
  - Mutational spectrum analysis
  - Generation of P812S spike mutants
  - Molecular dynamics simulations
  - Computing backbone rotamer shift with respect to WT
  - Pseudovirus preparation
  - Standardization of virus input by reverse transcriptase activity using SYBR green-based product enhanced RT-PCR assay (SG-PERT)
  - Western blot analysis
  - EC50 of entry inhibitors in A549-ACE2-TMPRSS2 cells with PV
  - Cell-cell fusion assay
  - Flow cytometry
  - Vaccinee serum and monoclonal antibody pseudotype virus (PV) neutralization assay
- QUANTIFICATION AND STATISTICAL ANALYSIS

SUPPLEMENTAL INFORMATION

Supplemental information can be found online at https://doi.org/10.1016/j.celrep.2025.116135.

Received: February 27, 2025
Revised: June 25, 2025
Accepted: July 20, 2025
Published: August 13, 2025

REFERENCES

1. Willett, B.J., Grove, J., MacLean, O.A., Wilkie, C., De Lorenzo, G., Furnon, W., Cantoni, D., Scott, S., Logan, N., Ashraf, S., et al. (2022). SARS-CoV-2 Omicron is an immune escape variant with an altered cell entry pathway. Nat. Microbiol. *7*, 1161–1179. https://doi.org/10.1038/s41564-022-01143-7.

2. Meng, B., Abdullahi, A., Ferreira, I.A.T.M., Goonawardane, N., Saito, A., Kimura, I., Yamasoba, D., Gerber, P.P., Fatihi, S., Rathore, S., et al. (2022). Altered TMPRSS2 usage by SARS-CoV-2 Omicron impacts infectivity and fusogenicity. Nature *603*, 706–714. https://doi.org/10.1038/s41586-022-04474-x.

3. Tamura, T., Ito, J., Uriu, K., Zahradnik, J., Kida, I., Anraku, Y., Nasser, H., Shofa, M., Oda, Y., Lytras, S., et al. (2023). Virological characteristics of the SARS-CoV-2 XBB variant derived from recombination of two Omicron subvariants. Nat. Commun. *14*, 2800. https://doi.org/10.1038/s41467-023-38435-3.

4. El Moussaoui, M., Bontems, S., Meex, C., Hayette, M.-P., Lejeune, M., Hong, S.L., Dellicour, S., Moutschen, M., Cambisano, N., Renotte, N., et al. (2024). Intrahost evolution leading to distinct lineages in the upper and lower respiratory tracts during SARS-CoV-2 prolonged infection. Virus Evolution *10*, veae073. https://doi.org/10.1093/ve/veae073.

5. Wang, Y., Wang, D., Zhang, L., Sun, W., Zhang, Z., Chen, W., Zhu, A., Huang, Y., Xiao, F., Yao, J., et al. (2021). Intra-host variation and evolutionary dynamics of SARS-CoV-2 populations in COVID-19 patients. Genome Med. *13*, 30. https://doi.org/10.1186/s13073-021-00847-5.

6. Van Cleemput, J., van Snippenberg, W., Lambrechts, L., Dendooven, A., D'Onofrio, V., Couck, L., Trypsteen, W., Vanrusselt, J., Theuns, S., Vereecke, N., et al. (2021). Organ-specific genome diversity of replication-competent SARS-CoV-2. Nat. Commun. *12*, 6612. https://doi.org/10.1038/s41467-021-26884-7.

7. Buckland, M.S., Galloway, J.B., Fhogartaigh, C.N., Meredith, L., Provine, N.M., Bloor, S., Ogbe, A., Zelek, W.M., Smielewska, A., Yakovleva, A., et al. (2020). Treatment of COVID-19 with remdesivir in the absence of humoral immunity: a case report. Nat. Commun. *11*, 6385. https://doi.org/10.1038/s41467-020-19761-2.

8. Voloch, C.M., da Silva Francisco, R., Jr., de Almeida, L.G.P., Brustolini, O. J., Cardoso, C.C., Gerber, A.L., Guimarães, A.P.d.C., Leitão, I.d.C., Mariani, D., Ota, V.A., et al. (2021). Intra-host evolution during SARS-CoV-2 prolonged infection. Virus Evol. *7*, veab078. https://doi.org/10.1093/ve/veab078.

9. Al Khatib, H.A., Benslimane, F.M., Elbashir, I.E., Coyle, P.V., Al Maslamani, M.A., Al-Khal, A., Al Thani, A.A., and Yassine, H.M. (2020). Within-Host Diversity of SARS-CoV-2 in COVID-19 Patients With Variable Disease Severities. Front. Cell. Infect. Microbiol. *10*, 575613. https://doi.org/10.3389/fcimb.2020.575613.

10. Rueca, M., Bartolini, B., Gruber, C.E.M., Piralla, A., Baldanti, F., Giombini, E., Messina, F., Marchioni, L., Ippolito, G., Di Caro, A., and Capobianchi, M.R. (2020). Compartmentalized Replication of SARS-Cov-2 in Upper vs. Lower Respiratory Tract Assessed by Whole Genome Quasispecies Analysis. Microorganisms *8*, 1302. https://doi.org/10.3390/microorganisms8091302.

11. Wolter, N., Jassat, W., Walaza, S., Welch, R., Moultrie, H., Groome, M., Amoako, D.G., Everatt, J., Bhiman, J.N., Scheepers, C., et al. (2022). Early assessment of the clinical severity of the SARS-CoV-2 omicron variant in South Africa: a data linkage study. Lancet *399*, 437–446. https://doi.org/10.1016/S0140-6736(22)00017-4.

12. Dufloo, J., and Sanjuán, R. (2024). Temperature impacts SARS-CoV-2 spike fusogenicity and evolution. mBio *15*, e03360-23. https://doi.org/10.1128/mbio.03360-23.

13. Kemp, S.A., Collier, D.A., Datir, R.P., Ferreira, I.A.T.M., Gayed, S., Jahun, A., Hosmillo, M., Rees-Spear, C., Mlcochova, P., Lumb, I.U., et al. (2021). SARS-CoV-2 evolution during treatment of chronic infection. Nature *592*, 277–282. https://doi.org/10.1038/s41586-021-03291-y.

14. Harari, S., Tahor, M., Rutsinsky, N., Meijer, S., Miller, D., Henig, O., Halutz, O., Levytskyi, K., Ben-Ami, R., Adler, A., et al. (2022). Drivers of adaptive evolution during chronic SARS-CoV-2 infections. Nat. Med. *28*, 1501–1508. https://doi.org/10.1038/s41591-022-01882-4.

15. Ruis, C., Peacock, T.P., Polo, L.M., Masone, D., Alvarez, M.S., Hinrichs, A. S., Turakhia, Y., Cheng, Y., McBroome, J., Corbett-Detig, R., et al. (2023). A lung-specific mutational signature enables inference of viral and bacterial respiratory niche. Microb. Genom. *9*, mgen001018. https://doi.org/10.1099/mgen.0.001018.

16. Weigang, S., Fuchs, J., Zimmer, G., Schnepf, D., Kern, L., Beer, J., Luxenburger, H., Ankerhold, J., Falcone, V., Kemming, J., et al. (2021). Within-host evolution of SARS-CoV-2 in an immunosuppressed COVID-19 patient as a source of immune escape variants. Nat. Commun. *12*, 6405. https://doi.org/10.1038/s41467-021-26602-3.

17. Choi, B., Choudhary, M.C., Regan, J., Sparks, J.A., Padera, R.F., Qiu, X., Solomon, I.H., Kuo, H.H., Boucau, J., Bowman, K., et al. (2020).

CellPress
OPEN ACCESS

Persistence and Evolution of SARS-CoV-2 in an Immunocompromised Host. N. Engl. J. Med. *383*, 2291–2293. https://doi.org/10.1056/nejmc2031364.

18. Tartof, S.Y., Slezak, J.M., Puzniak, L., Hong, V., Xie, F., Ackerson, B.K., Valluri, S.R., Jodar, L., and McLaughlin, J.M. (2022). Immunocompromise and durability of BNT162b2 vaccine against severe outcomes due to omicron and delta variants. Lancet Respir. Med. *10*, e61–e62. https://doi.org/10.1016/S2213-2600(22)00170-9.
19. Tartof, S.Y., Slezak, J.M., Puzniak, L., Hong, V., Frankland, T.B., Ackerson, B.K., Takhar, H.S., Ogun, O.A., Simmons, S.R., Zamparo, J.M., et al. (2022). Effectiveness of a third dose of BNT162b2 mRNA COVID-19 vaccine in a large US health system: A retrospective cohort study. Lancet Reg. Health. Am. *9*, 100198. https://doi.org/10.1016/j.lana.2022.100198.
20. Lee, A.R.Y.B., Wong, S.Y., Chai, L.Y.A., Lee, S.C., Lee, M.X., Muthiah, M. D., Tay, S.H., Teo, C.B., Tan, B.K.J., Chan, Y.H., et al. (2022). Efficacy of covid-19 vaccines in immunocompromised patients: systematic review and meta-analysis. BMJ *376*, e068632. https://doi.org/10.1136/bmj-2021-068632.
21. Fibke, C.D., Joffres, Y., Tyson, J.R., Colijn, C., Janjua, N.Z., Fjell, C., Prystajecky, N., Jassem, A., and Sbihi, H. (2022). Spike Mutation Profiles Associated With SARS-CoV-2 Breakthrough Infections in Delta Emerging and Predominant Time Periods in British Columbia, Canada. Front. Public Health *10*, 915363. https://doi.org/10.3389/fpubh.2022.915363.
22. Bussani, R., Schneider, E., Zentilin, L., Collesi, C., Ali, H., Braga, L., Volpe, M.C., Colliva, A., Zanconati, F., Berlot, G., et al. (2020). Persistence of viral RNA, pneumocyte syncytia and thrombosis are hallmarks of advanced COVID-19 pathology. EBioMedicine *61*, 103104. https://doi.org/10.1016/j.ebiom.2020.103104.
23. Braga, L., Ali, H., Secco, I., Chiavacci, E., Neves, G., Goldhill, D., Penn, R., Jimenez-Guardeño, J.M., Ortega-Prieto, A.M., Bussani, R., et al. (2021). Drugs that inhibit TMEM16 proteins block SARS-CoV-2 spike-induced syncytia. Nature *594*, 88–93. https://doi.org/10.1038/s41586-021-03491-6.
24. Gangavarapu, K., Latif, A.A., Mullen, J.L., Alkuzweny, M., Hufbauer, E., Tsueng, G., Haag, E., Zeller, M., Aceves, C.M., Zaiets, K., et al. (2022). Outbreak.info genomic reports: scalable and dynamic surveillance of SARS-CoV-2 variants and mutations. Preprint at medRxiv. https://doi.org/10.1101/2022.01.27.22269965.
25. Khare, S., Gurry, C., Freitas, L., Schultz, M.B., Bach, G., Diallo, A., Akite, N., Ho, J., Lee, R.T., Yeo, W., et al. (2021). GISAID's Role in Pandemic Response. China CDC Wkly. *3*, 1049–1051. https://doi.org/10.46234/ccdcw2021.255.
26. Benton, D.J., Wrobel, A.G., Xu, P., Roustan, C., Martin, S.R., Rosenthal, P. B., Skehel, J.J., and Gamblin, S.J. (2020). Receptor binding and priming of the spike protein of SARS-CoV-2 for membrane fusion. Nature *588*, 327–330. https://doi.org/10.1038/s41586-020-2772-0.
27. Hoffmann, M., Kleine-Weber, H., Schroeder, S., Krüger, N., Herrler, T., Erichsen, S., Schiergens, T.S., Herrler, G., Wu, N.-H., Nitsche, A., et al. (2020). SARS-CoV-2 Cell Entry Depends on ACE2 and TMPRSS2 and Is Blocked by a Clinically Proven Protease Inhibitor. Cell *181*, 271–280. https://doi.org/10.1016/j.cell.2020.02.052.
28. Bianchini, F., Crivelli, V., Abernathy, M.E., Guerra, C., Palus, M., Muri, J., Marcotte, H., Piralla, A., Pedotti, M., De Gasparo, R., et al. (2023). Human neutralizing antibodies to cold linear epitopes and subdomain 1 of the SARS-CoV-2 spike glycoprotein. Sci. Immunol. *8*, eade0958, *0*, eade0958. https://doi.org/10.1126/sciimmunol.ade0958.
29. Kemp, S.A., Cheng, M.T.K., Hamilton, W.L., Kamelian, K., Indian SARS-CoV-2 Genomics Consortium INSACOG; Singh, S., Rakshit, P., Agrawal, A., Illingworth, C.J.R., and Gupta, R.K. (2022). Transmission of B.1.617.2 Delta variant between vaccinated healthcare workers. Sci. Rep. *12*, 10492. https://doi.org/10.1038/s41598-022-14411-7.
30. Mlcochova, P., Kemp, S.A., Dhar, M.S., Papa, G., Meng, B., Ferreira, I.A.T. M., Datir, R., Collier, D.A., Albecka, A., Singh, S., et al. (2021). SARS-CoV-2 B.1.617.2 Delta variant replication and immune evasion. Nature *599*, 114–119. https://doi.org/10.1038/s41586-021-03944-y.
31. Peacock, T.P., Goldhill, D.H., Zhou, J., Baillon, L., Frise, R., Swann, O.C., Kugathasan, R., Penn, R., Brown, J.C., Sanchez-David, R.Y., et al. (2021). The furin cleavage site in the SARS-CoV-2 spike protein is required for transmission in ferrets. Nat. Microbiol. *6*, 899–909. https://doi.org/10.1038/s41564-021-00908-w.
32. Meng, B., Kemp, S.A., Papa, G., Datir, R., Ferreira, I.A.T.M., Marelli, S., Harvey, W.T., Lytras, S., Mohamed, A., Gallo, G., et al. (2021). Recurrent emergence of SARS-CoV-2 spike deletion H69/V70 and its role in the Alpha variant B.1.1.7. Cell Rep. *35*, 109292. https://doi.org/10.1016/j.celrep.2021.109292.
33. Hui, K.P.Y., Ng, K.-C., Ho, J.C.W., Yeung, H.-W., Ching, R.H.H., Gu, H., Chung, J.C.K., Chow, V.L.Y., Sit, K.-Y., Hsin, M.K.Y., et al. (2022). Replication of SARS-CoV-2 Omicron BA.2 variant in ex vivo cultures of the human upper and lower respiratory tract. EBioMedicine *83*, 104232. https://doi.org/10.1016/j.ebiom.2022.104232.
34. Krapp, C., Hotter, D., Gawanbacht, A., McLaren, P.J., Kluge, S.F., Stürzel, C.M., Mack, K., Reith, E., Engelhart, S., Ciuffi, A., et al. (2016). Guanylate Binding Protein (GBP) 5 Is an Interferon-Inducible Inhibitor of HIV-1 Infectivity. Cell Host Microbe *19*, 504–514. https://doi.org/10.1016/j.chom.2016.02.019.
35. Braun, E., Hotter, D., Koepke, L., Zech, F., Groß, R., Sparrer, K.M.J., Müller, J.A., Pfaller, C.K., Heusinger, E., Wombacher, R., et al. (2019). Guanylate-Binding Proteins 2 and 5 Exert Broad Antiviral Activity by Inhibiting Furin-Mediated Processing of Viral Envelope Proteins. Cell Rep. *27*, 2092–2104. https://doi.org/10.1016/j.celrep.2019.04.063.
36. Mesner, D., Reuschl, A.-K., Whelan, M.V.X., Bronzovich, T., Haider, T., Thorne, L.G., Ragazzini, R., Bonfanti, P., Towers, G.J., and Jolly, C. (2023). SARS-CoV-2 evolution influences GBP and IFITM sensitivity. Proc. Natl. Acad. Sci. USA *120*, e2212577120. https://doi.org/10.1073/pnas.2212577120.
37. Li, X., Yuan, H., Li, X., and Wang, H. (2023). Spike protein mediated membrane fusion during SARS-CoV-2 infection. J. Med. Virol. *95*, e28212. https://doi.org/10.1002/jmv.28212.
38. Rajah, M.M., Bernier, A., Buchrieser, J., and Schwartz, O. (2022). The Mechanism and Consequences of SARS-CoV-2 Spike-Mediated Fusion and Syncytia Formation. J. Mol. Biol. *434*, 167280. https://doi.org/10.1016/j.jmb.2021.167280.
39. Planas, D., Staropoli, I., Michel, V., Lemoine, F., Donati, F., Prot, M., Porrot, F., Guivel-Benhassine, F., Jeyarajah, B., Brisebarre, A., et al. (2024). Distinct evolution of SARS-CoV-2 Omicron XBB and BA.2.86/JN.1 lineages combining increased fitness and antibody evasion. Nat. Commun. *15*, 2254. https://doi.org/10.1038/s41467-024-46490-7.
40. Dacon, C., Tucker, C., Peng, L., Lee, C.-C.D., Lin, T.-H., Yuan, M., Cong, Y., Wang, L., Purser, L., Williams, J.K., et al. (2022). Broadly neutralizing antibodies target the coronavirus fusion peptide. Science *377*, 728–735. https://doi.org/10.1126/science.abq3773.
41. Low, J.S., Jerak, J., Tortorici, M.A., McCallum, M., Pinto, D., Cassotta, A., Foglierini, M., Mele, F., Abdelnabi, R., Weynand, B., et al. (2022). ACE2-binding exposes the SARS-CoV-2 fusion peptide to broadly neutralizing coronavirus antibodies. Science *377*, 735–742. https://doi.org/10.1126/science.abq2679.
42. Chen, Y., Zhao, X., Zhou, H., Zhu, H., Jiang, S., and Wang, P. (2023). Broadly neutralizing antibodies to SARS-CoV-2 and other human coronaviruses. Nat. Rev. Immunol. *23*, 189–199. https://doi.org/10.1038/s41577-022-00784-3.
43. Harvey, W.T., Carabelli, A.M., Jackson, B., Gupta, R.K., Thomson, E.C., Harrison, E.M., Ludden, C., Reeve, R., and Rambaut, A.; COVID-19 Genomics UK COG-UK Consortium (2021). SARS-CoV-2 variants, spike mutations and immune escape. Nat. Rev. Microbiol. *19*, 409–424. https://doi.org/10.1038/s41579-021-00573-0.
44. Naldini, L., Blömer, U., Gage, F.H., Trono, D., and Verma, I.M. (1996). Efficient transfer, integration, and sustained long-term expression of the

transgene in adult rat brains injected with a lentiviral vector. Proc. Natl. Acad. Sci. USA *93*, 11382–11388. https://doi.org/10.1073/pnas.93.21.11382.

45. Collier, D.A., Ferreira, I.A.T.M., Kotagiri, P., Datir, R.P., Lim, E.Y., Touizer, E., Meng, B., Abdullahi, A., and CITIID-NIHR BioResource COVID-19 Collaboration; and Elmer, A. (2021). Age-related immune response heterogeneity to SARS-CoV-2 vaccine BNT162b2. Nature *596*, 417–422. https://doi.org/10.1038/s41586-021-03739-1.
46. Collier, D.A., De Marco, A., Ferreira, I.A.T.M., Meng, B., Datir, R.P., Walls, A.C., Kemp, S.A., Bassi, J., Pinto, D., Silacci-Fregni, C., et al. (2021). Sensitivity of SARS-CoV-2 B.1.1.7 to mRNA vaccine-elicited antibodies. Nature *593*, 136–141. https://doi.org/10.1038/s41586-021-03412-7.
47. Lindemann, J., Leiacker, R., Rettinger, G., and Keck, T. (2002). Nasal mucosal temperature during respiration. Clin. Otolaryngol. *27*, 135–139. https://doi.org/10.1046/j.1365-2273.2002.00544.x.
48. Barnes, C.O., Jette, C.A., Abernathy, M.E., Dam, K.-M.A., Esswein, S.R., Gristick, H.B., Malyutin, A.G., Sharaf, N.G., Huey-Tubman, K.E., Lee, Y.E., et al. (2020). SARS-CoV-2 neutralizing antibody structures inform therapeutic strategies. Nature *588*, 682–687. https://doi.org/10.1038/s41586-020-2852-1.
49. Cox, M., Peacock, T.P., Harvey, W.T., Hughes, J., Wright, D.W., COVID-19 Genomics UK COG-UK Consortium; Willett, B.J., Thomson, E., Gupta, R.K., and Peacock, S.J. (2023). SARS-CoV-2 variant evasion of monoclonal antibodies based on in vitro studies. Nat. Rev. Microbiol. *21*, 112–124. https://doi.org/10.1038/s41579-022-00809-7.
50. Meng, B., Datir, R., Choi, J., CITIID-NIHR Bioresource COVID-19 Collaboration; Bradley, J.R., Smith, K.G.C., Lee, J.H., Gupta, R.K., Dougan, G., and Hess, C. (2022). SARS-CoV-2 spike N-terminal domain modulates TMPRSS2-dependent viral entry and fusogenicity. Cell Rep. *40*, 111220. https://doi.org/10.1016/j.celrep.2022.111220.
51. Ng, K.W., Faulkner, N., Finsterbusch, K., Wu, M., Harvey, R., Hussain, S., Greco, M., Liu, Y., Kjaer, S., Swanton, C., et al. (2022). SARS-CoV-2 S2–targeted vaccination elicits broadly neutralizing antibodies. Sci. Transl. Med. *14*, eabn3715. https://doi.org/10.1126/scitranslmed.abn3715.
52. Schmidt, F., Weisblum, Y., Muecksch, F., Hoffmann, H.-H., Michailidis, E., Lorenzi, J.C.C., Mendoza, P., Rutkowska, M., Bednarski, E., Gaebler, C., et al. (2020). Measuring SARS-CoV-2 neutralizing antibody activity using pseudotyped and chimeric viruses. J. Exp. Med. *217*, e20201181. https://doi.org/10.1084/jem.20201181.
53. Li, H. (2018). Minimap2: pairwise alignment for nucleotide sequences. Bioinformatics *34*, 3094–3100. https://doi.org/10.1093/bioinformatics/bty191.
54. Li, H., Handsaker, B., Wysoker, A., Fennell, T., Ruan, J., Homer, N., Marth, G., Abecasis, G., and Durbin, R.; 1000 Genome Project Data Processing Subgroup (2009). The Sequence Alignment/Map format and SAMtools. Bioinformatics *25*, 2078–2079. https://doi.org/10.1093/bioinformatics/btp352.
55. Danecek, P., Bonfield, J.K., Liddle, J., Marshall, J., Ohan, V., Pollard, M.O., Whitwham, A., Keane, T., McCarthy, S.A., Davies, R.M., and Li, H. (2021). Twelve years of SAMtools and BCFtools. GigaScience *10*, giab008. https://doi.org/10.1093/gigascience/giab008.
56. Cingolani, P., Platts, A., Wang, L.L., Coon, M., Nguyen, T., Wang, L., Land, S.J., Lu, X., and Ruden, D.M. (2012). A program for annotating and predicting the effects of single nucleotide polymorphisms, SnpEff: SNPs in the genome of Drosophila melanogaster strain w1118. Fly (Austin) *6*, 80–92. https://doi.org/10.4161/fly.19695 iso-2; iso-3. .
57. O'Toole, Á., Scher, E., Underwood, A., Jackson, B., Hill, V., McCrone, J.T., Colquhoun, R., Ruis, C., Abu-Dahab, K., Taylor, B., et al. (2021). Assignment of epidemiological lineages in an emerging pandemic using the pangolin tool. Virus Evol. *7*, veab064. https://doi.org/10.1093/ve/veab064.
58. Minh, B.Q., Schmidt, H.A., Chernomor, O., Schrempf, D., Woodhams, M.D., von Haeseler, A., and Lanfear, R. (2020). IQ-TREE 2: New Models and Efficient Methods for Phylogenetic Inference in the Genomic Era. Mol. Biol. Evol. *37*, 1530–1534. https://doi.org/10.1093/molbev/msaa015.
59. Kosakovsky Pond, S.L., and Frost, S.D.W. (2005). Not so different after all: a comparison of methods for detecting amino acid sites under selection. Mol. Biol. Evol. *22*, 1208–1222. https://doi.org/10.1093/molbev/msi105.
60. Amodio, E., Genovese, D., Fallucca, A., Ferro, P., Sparacia, B., D'Azzo, L., Fertitta, A., Maida, C.M., and Vitale, F. (2022). Clinical Severity in Different Waves of SARS-CoV-2 Infection in Sicily: A Model of Smith's "Law of Declining Virulence" from Real-World Data. Viruses *15*, 125. https://doi.org/10.3390/v15010125.
61. Lan, J., Ge, J., Yu, J., Shan, S., Zhou, H., Fan, S., Zhang, Q., Shi, X., Wang, Q., Zhang, L., and Wang, X. (2020). Structure of the SARS-CoV-2 spike receptor-binding domain bound to the ACE2 receptor. Nature *581*, 215–220. https://doi.org/10.1038/s41586-020-2180-5.
62. Kadam, S.B., Sukhramani, G.S., Bishnoi, P., Pable, A.A., and Barvkar, V.T. (2021). SARS-CoV-2, the pandemic coronavirus: Molecular and structural insights. J. Basic Microbiol. *61*, 180–202. https://doi.org/10.1002/jobm.202000537.
63. Zhang, J., Xiao, T., Cai, Y., and Chen, B. (2021). Structure of SARS-CoV-2 spike protein. Curr. Opin. Virol. *50*, 173–182. https://doi.org/10.1016/j.coviro.2021.08.010.
64. Pettersen, E.F., Goddard, T.D., Huang, C.C., Meng, E.C., Couch, G.S., Croll, T.I., Morris, J.H., and Ferrin, T.E. (2021). UCSF ChimeraX: Structure visualization for researchers, educators, and developers. Protein Sci. *30*, 70–82. https://doi.org/10.1002/pro.3943.
65. Ruis, C., Tonkin-Hill, G., Floto, R.A., and Parkhill, J. (2023). Calculating and applying pathogen mutational spectra using MutTui. Preprint at bioRxiv. https://doi.org/10.1101/2023.06.15.545111.
66. Abraham, M.J., Murtola, T., Schulz, R., Páll, S., Smith, J.C., Hess, B., and Lindahl, E. (2015). GROMACS: High performance molecular simulations through multi-level parallelism from laptops to supercomputers. SoftwareX *1–2*, 19–25. https://doi.org/10.1016/j.softx.2015.06.001.
67. Schrödinger, LLC (2015). The PyMOL Molecular Graphics System, Version 1.8.
68. Schrödinger, LLC (2015). The JyMOL Molecular Graphics Development Component, Version 1.8.
69. Schrödinger, LLC (2015). The AxPyMOL Molecular Graphics Plugin for Microsoft PowerPoint, Version 1.8.
70. Michaud-Agrawal, N., Denning, E.J., Woolf, T.B., and Beckstein, O. (2011). MDAnalysis: A toolkit for the analysis of molecular dynamics simulations. J. Comput. Chem. *32*, 2319–2327. https://doi.org/10.1002/jcc.21787.
71. Gowers, R., Linke, M., Barnoud, J., Reddy, T., Melo, M., Seyler, S., Domanski, J., Dotson, D., Buchoux, S., Kenney, I., and Beckstein, O. (2016). MDAnalysis: A Python Package for the Rapid Analysis of Molecular Dynamics Simulations. In, pp. 98–105. https://doi.org/10.25080/Majora-629e541a-00e.
72. Gupta, R.K., Kohli, A., McCormick, A.L., Towers, G.J., Pillay, D., and Parry, C.M. (2010). Full-length HIV-1 Gag determines protease inhibitor susceptibility within in-vitro assays. AIDS *24*, 1651–1655. https://doi.org/10.1097/QAD.0b013e3283398216.
73. Vermeire, J., Naessens, E., Vanderstraeten, H., Landi, A., Iannucci, V., Van Nuffel, A., Taghon, T., Pizzato, M., and Verhasselt, B. (2012). Quantification of Reverse Transcriptase Activity by Real-Time PCR as a Fast and Accurate Method for Titration of HIV, Lenti- and Retroviral Vectors. PLoS One *7*, e50859. https://doi.org/10.1371/journal.pone.0050859.

STAR★METHODS

KEY RESOURCES TABLE

REAGENT or RESOURCE	SOURCE	IDENTIFIER
Antibodies		
fp.006	Bianchini et al.[28]	
fp.007	Bianchini et al.[28]	
Goat Anti-Human ACE2 primary antibody	R&D Systems	Cat#AF933; RRID:AB_355722
Donkey Anti-Goat IgG H&L secondary antibody	Abcam	#ab150131; RRID:AB_2732857
Anti-Human TMPRSS2 Alexa Fluor 488-conjugated antibody	R&D Systems	Cat#FAB10723G; RRID:AB_3645473
rabbit anti-SARS-CoV-2 Spike antibody	Thermofisher Scientific	Cat# PA1-41165; RRID:AB_1087210, LOT:WE3286564
mouse anti-HIV-1 p55/p24	BEI Resources	Cat# ARP-3537; RRID:AB_3086785
mouse anti-Spike monoclonal	GeneTex	Cat# GTX632604; RRID:AB_2864418; clone 1A9
mouse anti-HA-tag	Biolegend	Cat# 901516; RRID:AB_2820200; clone 16B12
rabbit anti-p24	CFAR	Cat#ARP432
mouse Anti-Alpha-Tubulin	Sigma-Aldrich	Cat#MABT205; RRID:AB_11204167; cloneDM1A
Bacterial and virus strains		
XL10-Gold Ultracompetent Cells	Agilent	Cat#200314
Biological samples		
Human sera		
Chemicals, peptides, and recombinant proteins		
E64d	Tocris	Cat#4545
Camostat	Sigma-Aldrich	Cat#SML0057
Fugene HD Transfection Reagent	Promega	Cat#E2311
Fugene 6 Transfection Reagent	Promega	Cat#E2691
Cell lyisis buffer	Cell Signaling Technology	Cat#9803
Critical commercial assays		
Bright-Glo	Promega	Cat#E2650
QuikChange Lightning	Agilent	Cat#210518
QuantiTect SYBR Green PCR Kit	Qiagen	Cat#204143
Clarity Western ECL Substrate	Bio-Rad	Cat#1705061
Amersham ECL Prime	Cytivia	Cat#RPN2232
Experimental models: Cell lines		
HEK293T	ATCC	Cat#CRL-3216
HEK293T-GFP11	Leo James	N/A
Vero-GFP1-10	Leo James	N/A
CaLu-3	Paul Lehner	N/A
HeLa-ACE2	James Voss	N/A
A549-ACE2/TMPRSS2	Massimo Palmarini	N/A
Caco-2	ATCC	HTB-37
Experimental models: Organisms/strains		
Human serum samples from SARS-CoV-2 AdV vaccine recipients	Collected at NIHR BioResource Center, Cambridge	
Oligonucleotides		
SARS-CoV-2_Spike_P812S_FWD:GCCCG ATCCTAGCAAGAGCAGCAAGCGGAGC	This paper	NA

(*Continued on next page*)

Continued		
REAGENT or RESOURCE	SOURCE	IDENTIFIER
SARS-CoV-2_Spike_P812S_REV:GCTCC GCTTGCTGCTCTTGCTAGGATCGGGC	This paper	NA
SARS-CoV-2_Spike_T95I_FWD:GGGTGT ACTTTGCCAGCATCGAGAAGTCCAACATC	This paper	NA
SARS-CoV-2_Spike_T95I_REV:GATGTTG GACTTCTCGATGCTGGCAAAGTACACCC	This paper	NA
Recombinant DNA		
Plasmid: p8.91	This paper	N/A
Plasmid: CSFLW	This paper	N/A
Plasmid: pcDNA3.1	Thermo Scientific, Invitrogen	Cat#V66020
Software and algorithms		
PRISM	Graphpad	https://www.graphpad.com/
R	Rstudio	https://www.r-project.org/
FlowJo	Treestar	N/A
PyMOL	Schrödinger, LLC, 2015	
mafft v7.525	Open source (conda)	
pangolin V4.3	Open source (conda)	2021.3
Scorpio V0.3.19	Open source (conda)	v.153k
IQ-Treee v2.2.5	Open source (conda)	
HYPHY	Open source (conda)	

EXPERIMENTAL MODEL AND STUDY PARTICIPANT DETAILS

This study was primarily a laboratory based study using spike-presenting pseudotyped virus (PV) with mutations generated by site-directed mutagenesis. We test sensitivity to vaccinee sera, monoclonal antibody, and drug inhibitors. We tested infectivity in a variety of model cell lines.

Sensitivity to antibodies in serum was tested using sera collected from BNT162b2 vaccinees as part of the Cambridge NIHR Bio-resource. These individuals were vaccinated with two doses of AZD1222. 9 women and 11 men were included in the study with a mean age of 66 years of age.

Ethical approval

Ethical approval for this study involving human samples was obtained from the East of England Research Ethics Committee review board (17/EE/0025). All participants provided written informed consent.

Cell culture

CaLu-3 (human lung epithelial cell line; a gift from Paul Lehner) cells were maintained in Eagle's minimum essential medium (MEM). HEK293T (CRL-3216; a human kidney epithelial cell line), HeLa-ACE2 (a human cervix epithelial cell line overexpressing ACE2; a gift from James Voss), and A549-ACE2/TMPRSS2 (a human lung epithelial cell line overexpressing ACE2 and TMPRSS2).

All cell medium were fortified with 10% fetal bovine serum (FBS) and 1% penicillin-streptomycin (PS). All cells were regularly tested and are mycoplasma free. Cells are cultured at 10% CO_2 at 37°C, and for the alternative temperature experiments, at 10% CO_2 and 32°C 39°C.

Clinical sample collection and sequencing

Sequencing of SARS-CoV-2 viral DNA extracted from serial samples from the LRT (sputum and endotracheal aspirate), and URT (throat and nasal swab) was described previously in Kemp et al.[13] In short, nucleic acid extraction was done from 500 μL sample using the easyMAG platform (Biomerieux). Sequencing amplicons were prepared with the ARTICnetwork v.3 protocol and diluted to 2 ng μL^{-1} and 25 μL (50 ng) were used as input for each library preparation reaction, before sequencing using the Illumina MiSeq platform.

METHOD DETAILS

Sequence data processing and analysis

Illumina sequencing data from Kemp et al. was re-analysed.[13] Bam files were downloaded from Kemp et al. (BioProject PRJNA682013), mapped to Wuhan-Hu-1 (NC_045512.2) by minimap v2.28 (arguments -ax sr),[53] sorted and indexed by samtools v1.20,[54] and variants were called by BCFtools v1.18 functions mpileup (minimum base quality q30), and call (arguments –multiallelic-caller –variants-only).[55] Mutations identified by BCFtools were filtered by a minimum depth cutoff of 20x before annotations by snpEff v5.2 using the publicly available NC_045512.2 database.[56]

Genetic diversity and viral load analysis

The genetic diversity of each sample was estimated using Shannon Entropy based on the frequency of each iSNV, assuming that all iSNVs are independent from each other.

$$H = -\sum_{i}^{n} P(i) log_e P(i)$$

Where *P(i)* is the iSNV percentage frequency at gene loci *i*.

Shannon Entropy of NT samples collected prior to day 93, NT samples collected on day 93 and after, and ETA samples collected on day 93 and after were compared using student's t-test. For Ct values, Welch's t test was performed.

Phylogenetic analysis and lineage assignment

Excluding duplicates, and low coverage sequences, we searched GISAID for all complete coverage SARS-CoV-2 sequences with the Spike_P812S substitution on the 7th April 2024. All sequence encoding Spike-S812 were aligned to MN908947.3 by mafft (v7.525, –6merpair –keeplength –addfragments), and then were assigned lineages using Pangolin (V4.3).[57] The major lineages were grouped by Scorpio (V0.3.19). We stratified the sequences based on their major lineage designations and then performed stratified random sampling with proportional allocation to major lineages to obtain a subset of 250 P812S-containing sequences using the R package Rsample v1.2.1.

We constructed a maximum-likelihood tree from patient X1 and the 250 sequence sample using IQ-Tree v2.2.5 employing the GTR+F+I model of nucleotide substitution (model with the lowest Bayesian Information Criterion identified by IQ-Tree ModelFinder) with clustering support assessed by 1000 rounds of ultrafast bootstrapping.[58] Slatkin-Maddison's compartmentalisation test was performed using HYPHY v2.5.73.

Signals of natural selection in the SARS-CoV-2 S-gene

Mutational sites found in the patient, the Alpha variant, and the Delta variant, together with sites of neutralisation escape mutations were chosen for natural selection analysis using the internal-branch fixed effects (IEFL) method implemented in HYPHY.[59] All globally sampled near-full length SARS-CoV-2 sequences are sampled between December 2019 and March 2022 were binned in three-monthly intervals (labeled according to the last month of sampling within a bin) and analyzed separately within the context of the entire Spike open reading frame: an analysis setup needed for both phylogenetic context and for informing the nucleotide and codon substitution models upon which the IEFL method relies. Only codon substitutions mapped to internal phylogenetic tree branches were considered in these analyses since mutations mapping to terminal branches would have likely experienced insufficient durations of selection to yield meaningful evolutionary information. The approximate dominant-variant era during the COVID-19 pandemic where indicated based on results from Amodio and colleagues.[60]

Structure analysis

Coordinates for the SARS-CoV-2 Spike trimer complex with two RBD down and one RBD up was from PDB: 6XM4-Chain A to C, and domains definition from multiple references were used.[28,61–63] Graphics describing the structures were made in UCSF Chimera X V1.8.[64]

Mutational spectrum analysis

We calculated the mutational spectrum of mutations within each patient sample using the mutation files generated above. The korimuto pipeline within MutTui v2.0.2[65] was run on each sample independently using NC_045512.2 as the reference genome. The proportion of G>T mutations within each sample was calculated as the number of G>T mutations divided by the total number of mutations.

Generation of P812S spike mutants

Amino acid substitutions were introduced into a codon-optimised pCDNA3.1_SARS-CoV-2 WT, Δ69/70, Delta **(B.1.617.2, EPI_ISL_2378732)**, and Omicron (**EPI_ISL_7418017**) Spike Plasmid as previously described,[2,13,30] using the QuikChange Lightning Site-Directed mutagenesis kit, as per manufacturer's protocol (Agilent Technologies).

Molecular dynamics simulations

The spike protein in '1-RBD-up' state in complex with ACE-2 was considered for molecular dynamics simulation. The D614G spike was considered as the wild type (WT) protein. Along with D614 spike, three different mutant systems were prepared – i) WT + P812S, ii) WT + D796H + del 69/70 and iii) WT + D796H + del 69/70 + P812S. The spike protein was glycosylated as given in Casalino et al., 2020. The systems were solvated with TIP3P water and further neutralized with the counter ions. All the simulations were carried out with Gromacs version 2021.3 incorporating the CHARMM36 all-atom force field.[66] A grid spacing of 0.16 nm was used along with the fourth-order cubic interpolation for Particle Mesh Ewald (PME) summation. The van der Waals cut-off was set to 1.2 nm. The systems were initially subjected to energy minimization with steepest descent method. Temperature and pressure were maintained at 310 K and 1 bar using V-rescale thermostat and Parrinello-Rahman barostat, respectively. The numerical integration of equation of motion was computed every 2 fs and the coordinates of the system were saved 100 ps. Each system was simulated for 1 μs. The structures were visualized and rendered with PyMOL.[67–69] All basic distance and RMSD/RMSF calculations were performed in Python with MDAnalysis library.[70,71]

Computing backbone rotamer shift with respect to WT

To track the residues that has changed their backbone dihedral conformations, we computed a comparative metric called rotamer shift. We discretized the observed backbone dihedral angle into *cis* and *trans* states as given in Singh et al., 2017. We quantify the rotamer shift for the residue R_i with the following equation.

$$Rotamer\ Shift_{R_i\ WT\ vs\ MUT} = \frac{\sum_{r=0}^{N=n(r)} \left| f(r)_{WT} - f(r)_{MUT} \right|}{N}$$

The r denotes the rotamer state of the dihedral. For backbone dihedrals, the rotamer states include Cis and Trans. Thus, N is the total number of rotamers for a dihedral. Each of the rotamer fractions f(r) (fraction of a rotamer r of a residue throughout the trajectory) is computed for WT and the mutant and subtracted. The rotamer shift is then defined as the average of absolute value of these differences in the rotamer fractions. The resulting shift varies from 0 (no changes in rotamer fractions) to 1 (complete change in rotamer fractions). We computed rotamer shift in ψ and ϕ angles for each residue. The maximum of two was finally considered to represent the dihedral change occurred at a residue. The dihedral angles were calculated with the gromacs command *gmx rama*. The rotamer assignment and the shift calculations were performed with the in-house Python script.

Pseudovirus preparation

Pseudovirus were prepared by transfecting HEK293T cells with 1μg of p8.91 HIV-1 gag-pol expression vector, 1.5 μg of pCSFLW, and 1μg of plasmid expressing the respective spike protein using a 3:1 ratio of Fugene HD transfection agent (Promega).[44,72] After 48 h of incubation at 10% CO_2, 37°C, viral supernatant was collected and filtered through a .45 μm filter and stored at −80°C until further use. The 50% tissue-culture infectious dose ($TCID_{50}$) of SARS-CoV-2 pseudovirus was determined using the Steady-Glo Luciferase assay system (Promega).

For GBP5 sensitivity assays, pseudoviruses were prepared as described previously.[36] In brief, HEK293T cells were transfected the indicated spike plasmids, p.8.91 and pCSLW in the presence of 40ng GBP5 vector[34,35] or a corresponding empty vector control using Fugene6 (Promega). After 48-72h, culture supernatants were collected and 0.45 μm filtered.

Standardization of virus input by reverse transcriptase activity using SYBR green-based product enhanced RT-PCR assay (SG-PERT)

The reverse transcriptase activity of pseudovirus preparations was determined by quantitative polymerase chain reaction (qPCR) using an SYBR green-based product-enhanced PCR assay (SG-PERT) as previously described.[73] Briefly, undiluted supernatants were lysed in a 1:1 ratio in a 2x lysis solution (40% glycerol v/v, 0.25% Trition X-100 v/v, 50 mM KCl, Ribolock RNase inhibitor 0.8 U/mL, TrisHCl 100mM, buffered to pH 7.4) for 10 min at room temperature.

Western blot analysis

For virus pellets, the filtered viral supernatant was centrifuged at 15,000 rpm for 120 min to pellet virions. For cell lysates, transfected HEK293T cells were resuspended in PBS, before centrifugation at 1500 rpm for 5 min. The cell pellet was then lysed in 400 μL cell lysis buffer (Cell Signaling Technology) and sonicated for 1 min.

All samples are further lysed and reduced in Laemmli reducing buffer (1M Tris-HCl (pH6.8), SDS, 100% glycerol, β-mercaptoethanol and bromophenol blue). This was followed by electrophoresis on SDS 4–12% Bis-Tris protein gels (Thermo Fisher Scientific) under reducing conditions. Proteins on the gel are transferred by electroblotting onto polyvinylidene difluoride (PVDF) membranes. The SARS-CoV-2 spike proteins and pseudotype virus capsid were visualized using a ChemiDoc MP imaging system (Biorad) or Odyssey Cxl Infrared Imager (Licor) using a rabbit anti-Spike polyclonal (Thermo Fisher Scientific, 1 in 4000), mouse anti-p24 monoclonal (BEI Resources, 1 in 3000), mouse anti-Spike monoclonal (GeneTex, 1:1000), mouse anti-HA-tag (Biolegend, 1:2000), rabbit anti-p24 (CFAR, 1:2000) and mouse anti-alpha-tubulin (Sigma, 1:1000).

Chemiluminescence was detected using Bio-Rad Clarity Western ECL Substrate (Cat#1705061), or Cytiva Amersham ECL Prime (Cat#RPN2232), and the band intensity is quantified using ImageJ (NIH).

EC50 of entry inhibitors in A549-ACE2-TMPRSS2 cells with PV

A549-ACE2/TMPRSS2 (A549-A2T2) cells were treated with either E64d (Tocris) or camostat (Sigma-Aldrich) for 2 h at each drug concentration before the addition of a comparable amount of input pseudotyped viruses (approximately 500,000 RLU). The cells were then left for 48 h before addition of lysis buffer + luciferase substrate (Promega) and read on a Glomax plate reader (Promega). Normalisation was performed using an inhouse script, and EC_{50} values were calculated using a nonlinear regression model of inhibitor dilution vs. normalized response with a variable slope in GraphPad Prism 10.

Cell-cell fusion assay

Cell-cell fusion assays were performed as previously described.[2] Briefly, Vero-GFP1-10 and HEK293T-GFP11 were mixed and seeded at a 1:1 ratio at 80% confluence in 48-well plates the previous day. Cells were co-transfected with 250 ng of spike expressing plasmids using Fugene 6 following the manufacturer's instructions (Promega). Images were acquired using the IncuCyte SX5 Live-Cell Analysis System (Sartorius). Cell-cell fusion was determined as the proportion of green area to the total phase area and was calculated with the IncuCyte analysis software (Sartorius).

Flow cytometry

After incubation in a 6-well plate (Corning), cells were dissociated using trypsin in their respective temperatures for 5–7 min before the addition of the cell culture medium to resuspend the cells. Cells were spun down before they were transferred into round-bottom 96-well plates and incubated with Live/Dead Fix Near IR (Thermo Fisher Scientific; #L34994; 1:1000 dilution) in PBS for 15 min at room temperature. Cells were then resuspended in 100μL of 4% paraformaldehyde (PFA) after two washes with PBS and incubated for 20 min at room temperature in the dark. After two washes in FACS buffer (PBS +2.5% FBS + 2mM EDTA), cells were stained with 100μL of Anti-Human TMPRSS2 Alexa Fluor 488-conjugated antibody (R&D Systems; #FAB10723G; 1:400 dilution) and Anti-Human ACE2 primary antibody (R&D Systems; #AF933; 1:400 dilution) overnight at 4°C. After two washes in FACS buffer, cells were resuspended in 100μL of Donkey Anti-Goat IgG H&L secondary antibody (Abcam; #ab150131; 1:1000 dilution) in FACS buffer for 1 h at 4°C in the dark. After two further washes in FACS buffer, the stained samples were then acquired on a LSRFortessa X-20 (BD Biosciences) and analysis was conducted on FlowJo (BD Biosciences).

Vaccinee serum and monoclonal antibody pseudotype virus (PV) neutralization assay

Previous work have shown that IC_{50} obtained from spike pseudotype assays quantitatively correlate with neutralisation activity measured using an authentic SARS-CoV-2 neutralization assay.[52]

Virus neutralisation assays were conducted on HeLa cells stably expressing ACE2 (HeLa-A2) using SARS-CoV-2 spike pseudotyped virus expressing firefly luciferase as previously described.[13]

Sera were first heat-inactivated at 55°C for an hour. Serial dilutions of sera or purified monoclonal antibodies are then incubated with pseudotyped virus for 1h at 37°C. Freshly trypsinized and quenched HEK293T-A2T2 or HeLa-A2 cells were subsequently added to each well. After 48h incubation at 37°C and 5% CO_2, luminescence was measured using a Bright-Glo luciferase assay system (Promega) and neutralisation was calculated relative to cell-only and cell-and-virus-only controls. Normalisation was performed using an inhouse script, and IC_{50} values were calculated using a nonlinear regression model of inhibitor dilution vs. normalized response with a variable slope in GraphPad Prism 10.

QUANTIFICATION AND STATISTICAL ANALYSIS

Experiments were done at least two times with two to four technical replicates. Relative luciferase units (RLU) were measured with a Glomax luminometer. Data were analyzed using GraphPad PRISM software (version 10) or Rstatix v0.7.2. Statistical tests are described in the figure legends along n, mean, and standard deviation/error, where appropriate. Significant differences are annotated as *$p < 0.05$; **$p < 0.01$; ***$p < 0.001$; ****$p < 0.0001$.

Cell Reports, Volume *44*

Supplemental information

Signatures of omicron-like adaptation

in early SARS-CoV-2 variants and chronic infection

Mark Tsz Kin Cheng, Mazharul Altaf, Jesu Castin, Ann-Kathrin Reuschl, Benjamin L. Sievers, Kimia Kamelian, Dejan Mesner, Rebecca B. Morse, Adam Abdullahi, Bo Meng, Kata Csiba, Cambridge NIHR Bioresource, Steven A. Kemp, Darren P. Martin, Clare Jolly, Christopher Ruis, Lipi Thukral, and Ravindra K. Gupta

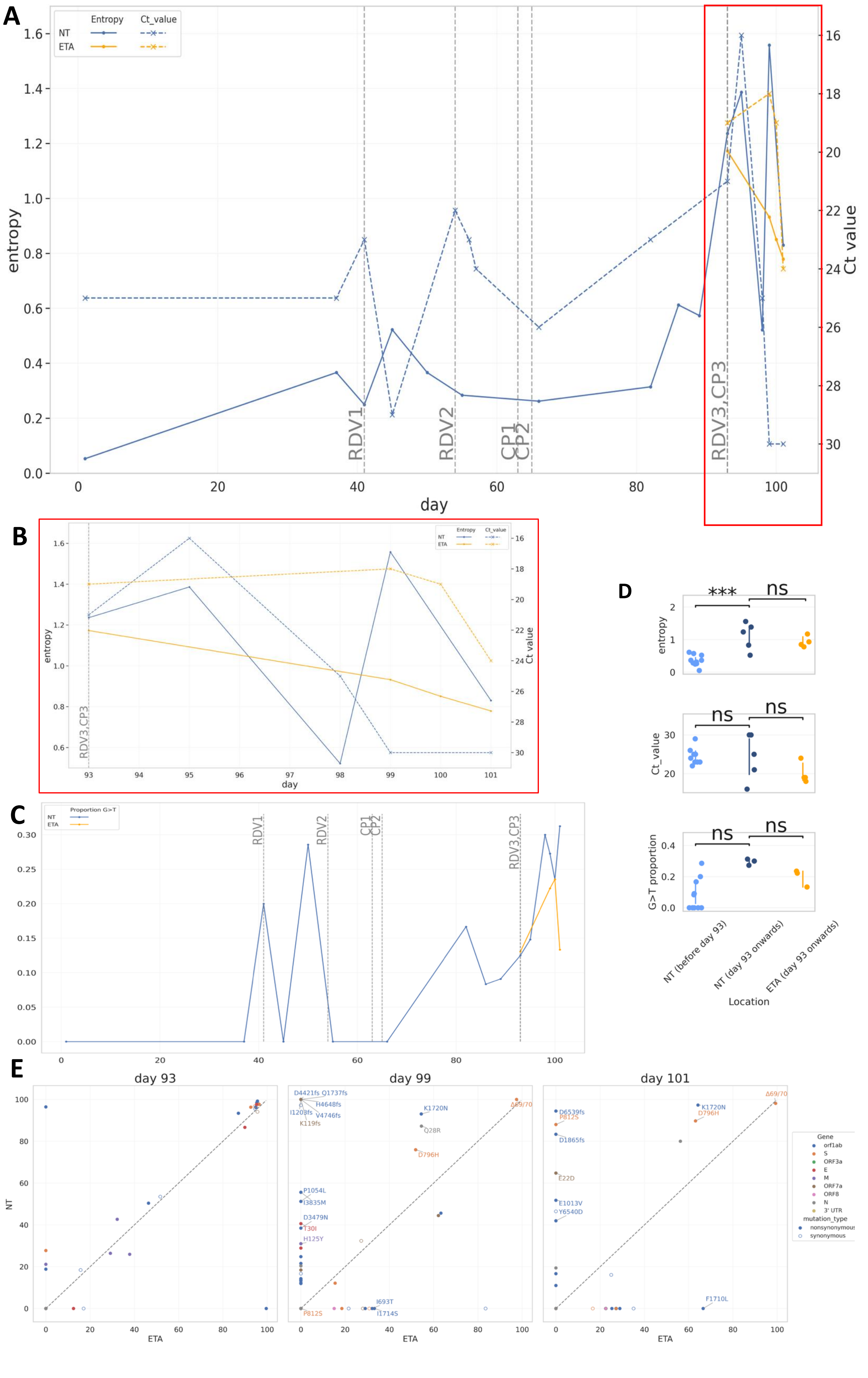

Supplementary Figure 1: Viral load and Intra-host evolution dynamics. A) Shannon's entropy (solid line, left y-axis) and Ct value (dotted line, right y-axis) of nose and throat (N+T, blue) and endotracheal aspirate (ETA, yellow) samples, zoomed in and rescaled from day 93 to day 101 (B). CP, convalescent plasma; RDV, Remdesivir. C) proportion of G to T mutation from day 1 to day 101. Day 93 and Day 95 were excluded as they are separated from the main clade. D) Dot plot (with 95% confidence interval) of Shannon's entropy (top) and Ct value (bottom) for NT samples from day 1-92, NT samples from day 93-101, and ETA samples from day 93 to 101. Student's t-test is performed for entropy whilst Welch correction was performed for Ct value in lieu of unequal variance. E) The percentage prevalence in NT plotted against ETA. Nonsynonymous mutations (filled) with >30% difference are annotated. The grey reference line represents equivalent prevalence between NT and ETA.

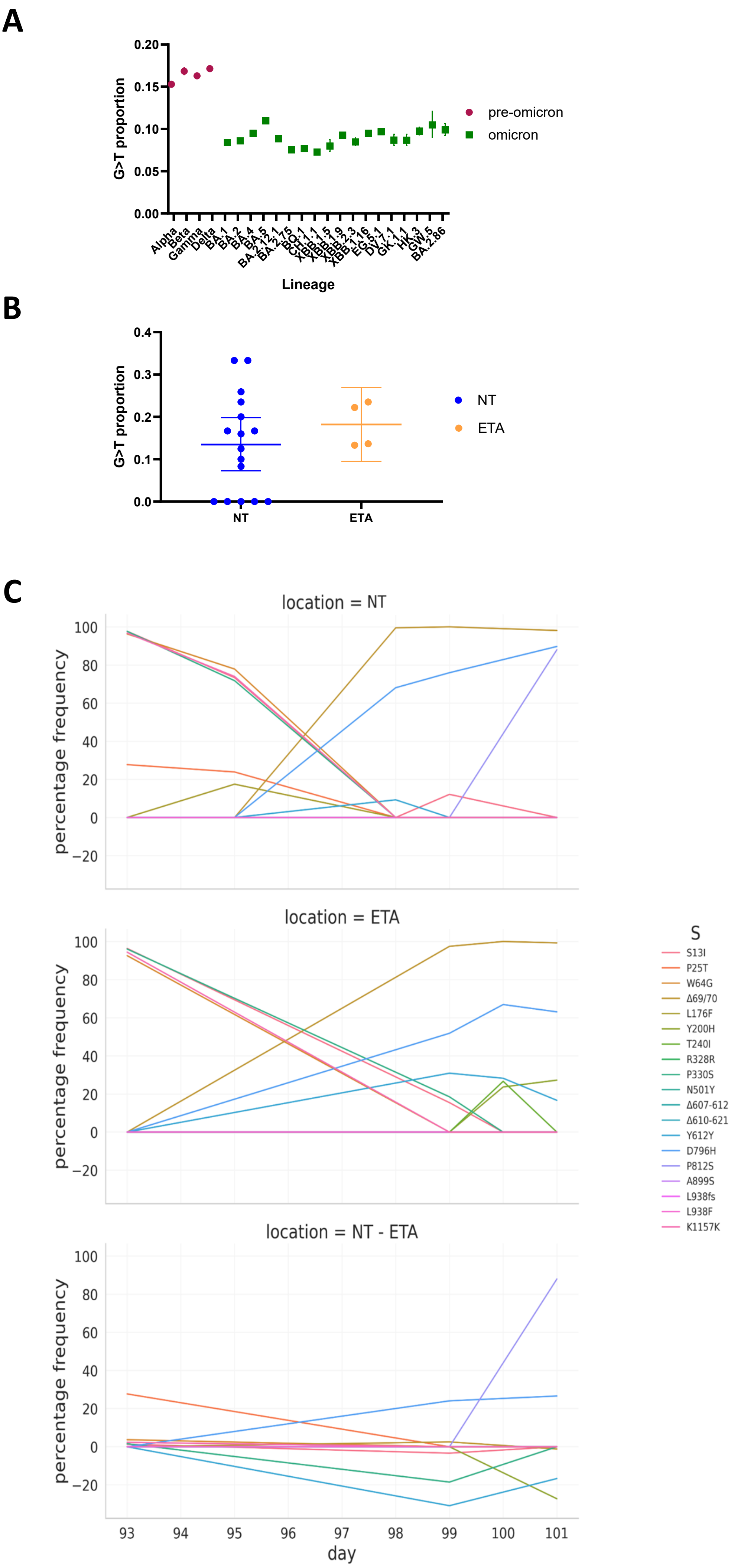

Supplementary Figure 2: Breakdown of mutational spectra and viral iSNV dynamics in the nasal and endotracheal compartments. The G to T proportion of A) globally circulating lineages in chronological order and B) of patient sample broken down by NT and ETA samples across all timepoints. C) Spike mutation frequency in N+T (top), ETA (middle), and absolute percentage difference (bottom) between day 93 and 101.

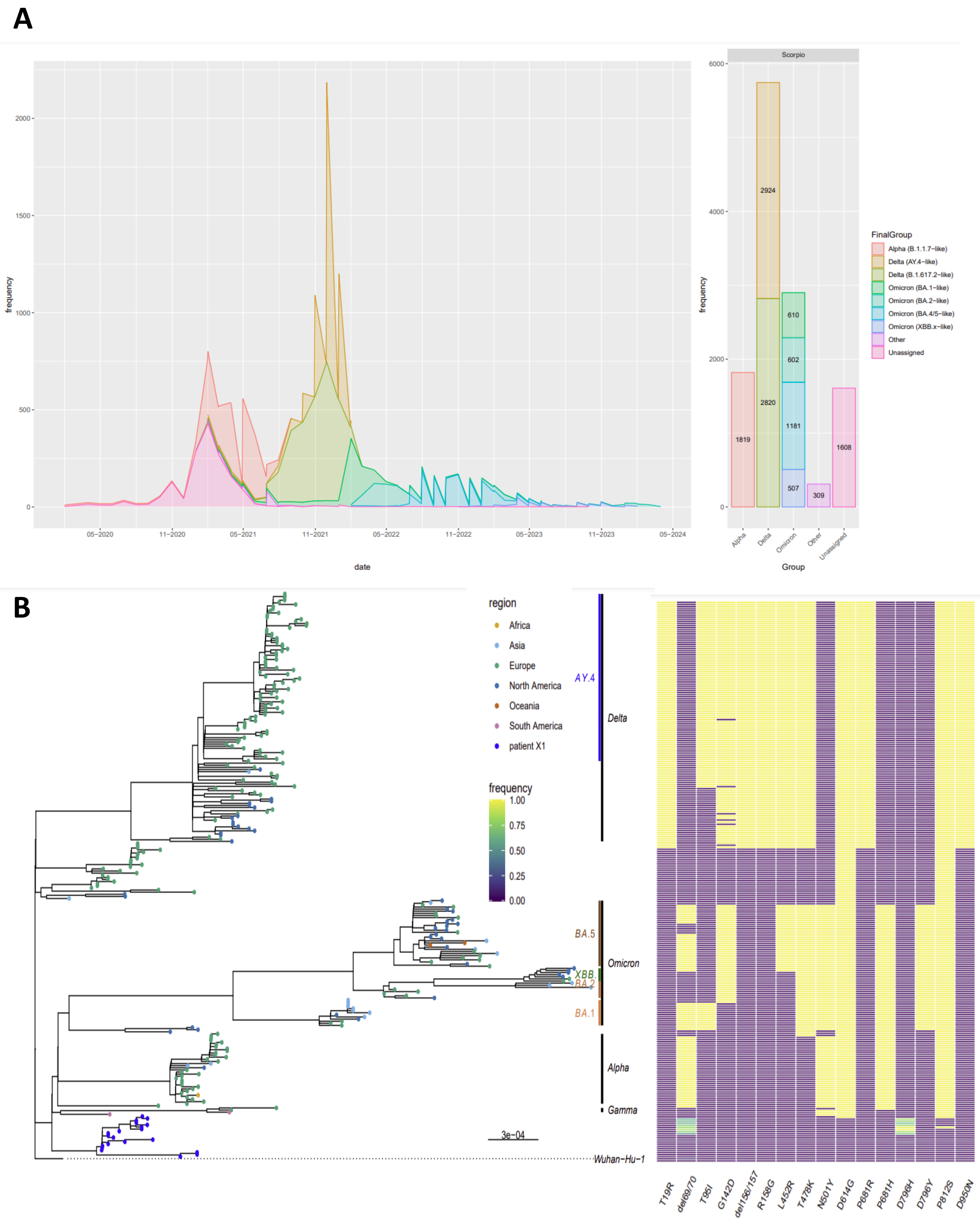

Supplementary Figure 3: Global Context of P812S containing SARS-CoV-2 global variants. A) The global prevalence of SARS-CoV-2 sequences with the S:P812S mutation in different variants defined by Scorpio as of 7th April 2024. B) Left panel - Maximum Likelihood tree (model: GTR+F+I) of patient X1 and 247 variant-stratified subsampled sequences. Tips are labelled by region. Right panel – Aligned heatmap of Spike mutations of all the subsampled sequences. For the subsampled GISAID sequences, yellow represent the presence and mauve the absence of the mutation at the consensus level.

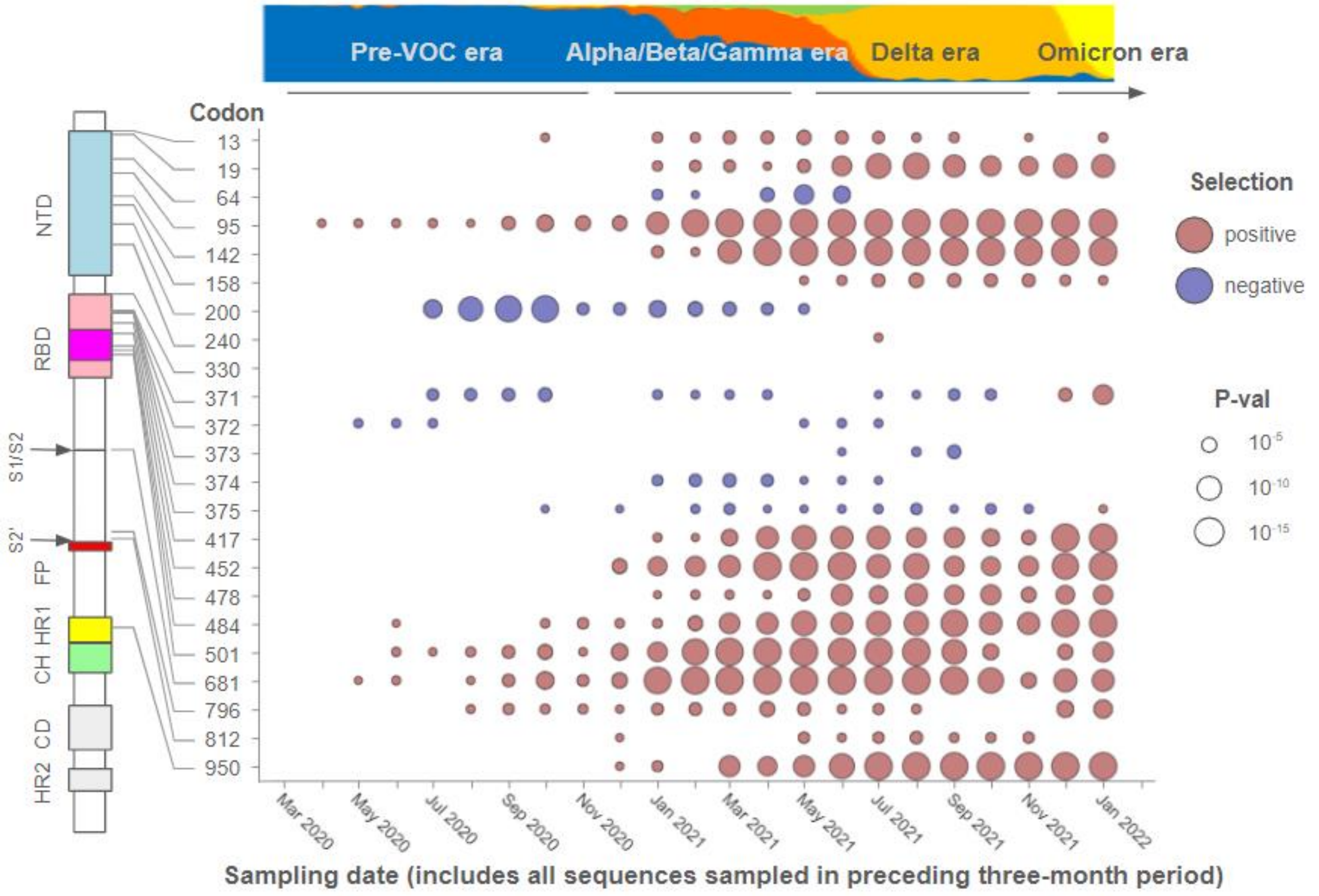

Supplementary Figure 4: Signals of natural selection at a sample of SARS-CoV-2 S-Gene codon sites during the early phases of the COVID-19 pandemic. Evidence of positive (red circles) and negative (blue circles) was detected using the IFEL method using all globally sampled near-full length SARS-CoV-2 sequences in the three months preceding the indicated sampling dates with the sizes of circles indicating the degree of statistical support for the detected selection signals (see key on the right). The approximate era during the COVID-19 pandemic (after Amodio et al) is indicated at the top. The non-variant of concern (VOC) lineages indicated in blue, the Alpha VOC in Red, the Beta VOC in dark orange, the Gamma VOC in green, the Delta VOC in orange and the Omicron VOC in yellow. The approximate location in the Spike protein of amino acids encoded by the various codon sites is indicated on the left: NTD = N-terminal domain; RBD = Receptor binding domain; FP = Fusion peptide; HR1 = Heptad repeat 1; CH = Central helix region, CD = Connector domain; HR2 = Heptad repeat 2.

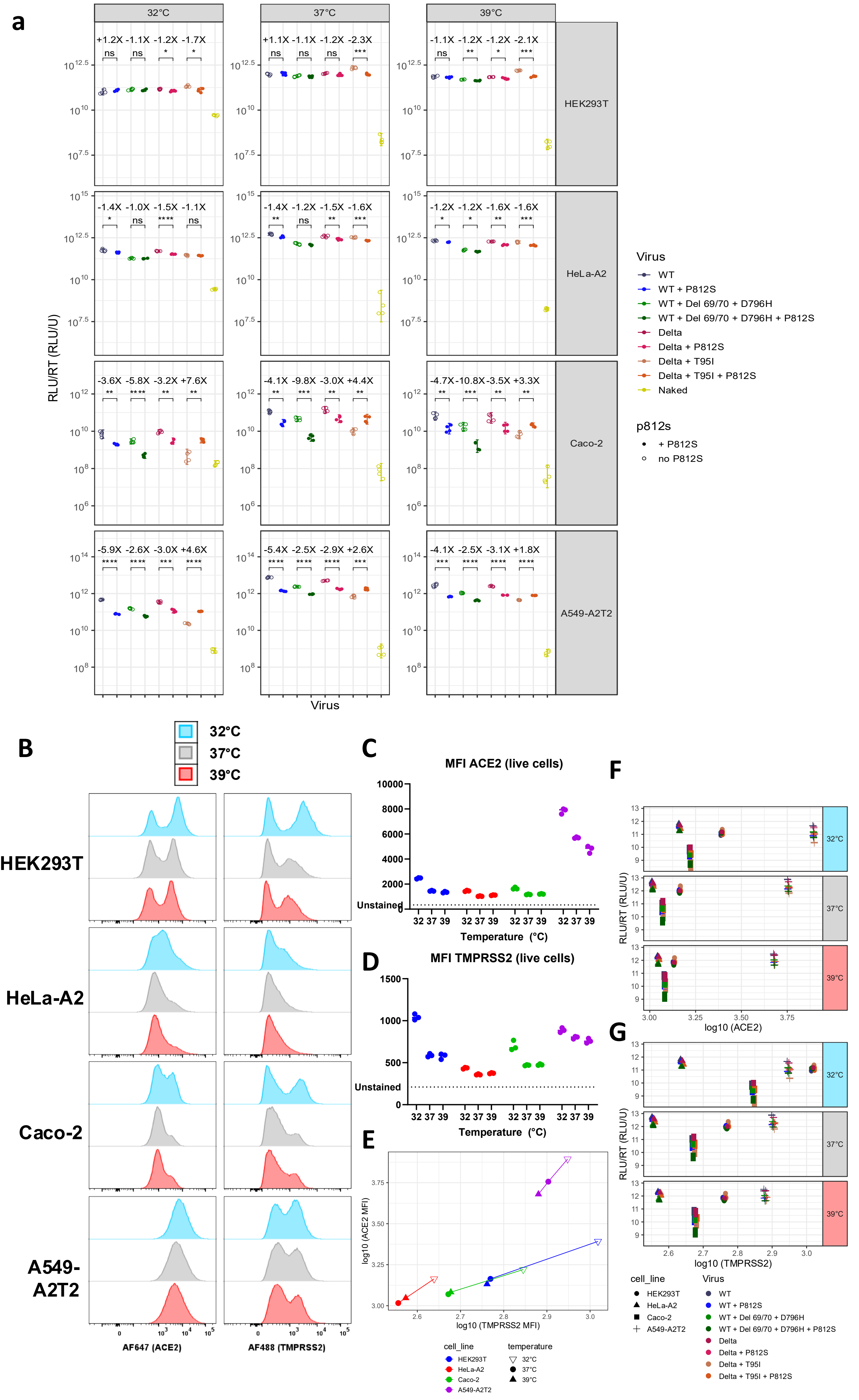

Supplementary Figure 5: P812S has a detrimental effect on cell entry in additional cell lines across 32°C, 37°C, and 39°C. A) RT-activity corrected infectivity of WT (Wuhan-Hu-1+D614G), WT+P812S, WT+Δ69/70+796H, WT+Δ69/70+796H+P812S, Delta, Delta+P812S, Delta+T95I, Delta +T95I+P812S, Naked (no spike) bearing PV introduced to cells of varying levels of ACE2 and TMPRSS levels pre-incubated at 32, 37, and 39 degrees. Data are supplemented by mean ± standard error of n = 4 replicates. Statistical analysis was performed using unpaired student's t-test with Benjamini-Hochberg correction on log-transformed values. B-E) ACE2 and TMPRSS2 surface expression of live HEK293T, HeLa-A2, CaCo-2, A549-A2T2 at 32°C, 37°C, and 39°C. b) densitogram of ACE2 and TMPRSS2 expression c,d) Mean fluorescence intensity of C) ACE2 and D) TMPRSS2 in HEK293T, HeLa-A2, CaCo-2, A549-A2T2. E) scatter plot of mean log10(ACE2) and log10(TMPRSS2) expression. F,G) RT-activity corrected infectivity of WT (Wuhan-Hu-1+D614G), WT+P812S, WT+Δ69/70+796H, WT+Δ69/70+796H+P812S, Delta, Delta+P812S, Delta+T95I, Delta +T95I+P812S plotted against log10 (ACE2) and log10 (TMPRSS2) at 32°C, 37°C, and 39°C. HEK293T, HeLa-A2, CaCo-2, A549-A2T2 are represented by shapes.

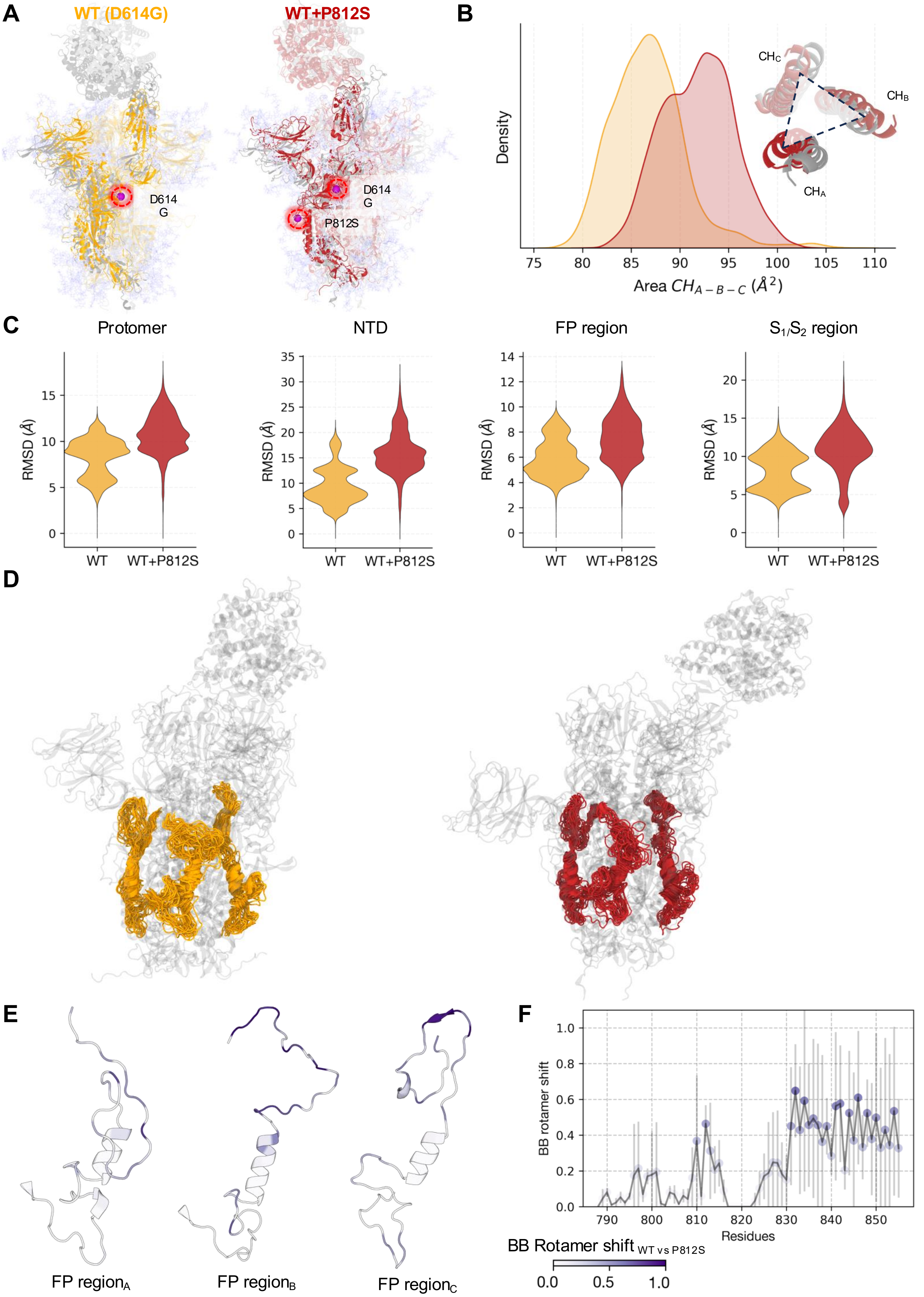

Supplementary Figure 6. The P812S mutant impacts the dynamics at both the S1/S2 and FP/S2' cleavage sites of Spike protein.
A) Snapshot of WT (D614G) and P812S spike trimers in complex with ACE-2. Both 0ns (grey) and 1μs timepoints superimposed on each other. B) The probability density plot of the triangle area formed by the centers of three CH domains is compared between WT and P812S mutant. The snapshot highlights the eagle-eye view of inter-CH orientation in WT (grey) and P812S mutant (dark red). C) The violin plots compare the distribution of RMSD calculated for the entire protomer, NTD, FP and S1/S2 regions respectively. D) The snapshots highlight the ensemble of FP conformations in WT (left) and P812S mutant (right). E) The backbone rotamer shift calculated between WT and P812S is mapped on the individual FP regions of the spike trimer. F) The corresponding rotamer shift values are plotted for FP residues. The values are averaged across three chains.

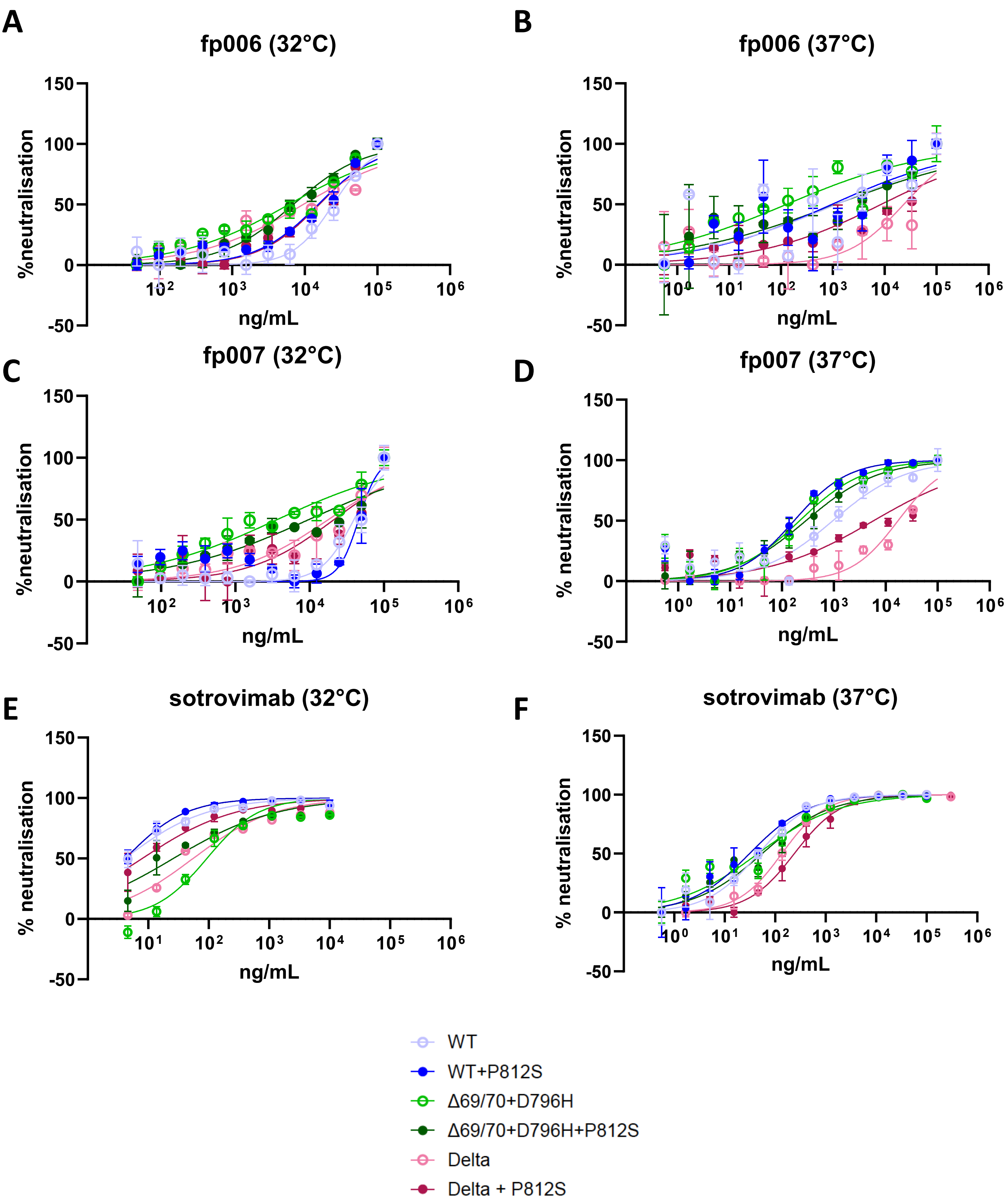

Supplementary Figure 7: Individual neutralisation curves for monoclonal antibodies fp006, fo007, and sotrovimab at 32'C and 37°C against SARS-CoV-2 spike mutants. Results relate to Figure are representative of 2 technical replicates Data points are representative of 2 technical replicates. Error bars represent SD.

Supplementary Tables

Supplementary Table 1: Lineage of P812S-containing sequences on GISAID

scorpio_call	Aggregate Group	Number of sequences	Percentage of total P812S-containing sequences
Alpha (B.1.1.7-like)	Alpha (B.1.1.7-like)	1818	14.68%
B.1.1.7-like+E484K	Alpha (B.1.1.7-like)	1	0.01%
Delta (AY.4-like)	Delta (AY.4-like)	2903	23.45%
Delta (AY.4.2-like)	Delta (AY.4-like)	21	0.17%
Delta (B.1.617.2-like)	Delta (B.1.617.2-like)	2820	22.78%
Omicron (BA.1-like)	Omicron (BA.1-like)	610	4.93%
Omicron (BA.2-like)	Omicron (BA.2-like)	602	4.86%
Omicron (BA.5-like)	Omicron (BA.4/5-like)	1127	9.10%
Omicron (BA.4-like)	Omicron (BA.4/5-like)	54	0.44%
Omicron (XBB.1-like)	Omicron (XBB.x-like)	210	1.70%
Omicron (XBB.1.5-like)	Omicron (XBB.x-like)	162	1.31%
Omicron (XBB-like)	Omicron (XBB.x-like)	109	0.88%
Omicron (XBB.1.16-like)	Omicron (XBB.x-like)	26	0.21%
Gamma (P.1-like)	Other	167	1.35%
A.23.1-like	Other	61	0.49%
Iota (B.1.526-like)	Other	20	0.16%
Epsilon (B.1.429-like)	Other	18	0.15%
Beta (B.1.351-like)	Other	16	0.13%

Epsilon (B.1.427-like)	Other	7	0.06%
Mu (B.1.621-like)	Other	7	0.06%
Lambda (C.37-like)	Other	6	0.05%
B.1.617.1-like	Other	4	0.03%
Eta (B.1.525-like)	Other	1	0.01%
Theta (P.3-like)	Other	1	0.01%
Zeta (P.2-like)	Other	1	0.01%
Unassigned	Unassigned	1590	12.84%
Probable Omicron (Unassigned)	Unassigned	10	0.08%
Omicron (Unassigned)	Unassigned	8	0.06%
	Total	12380	

All SARS-CoV-2 isolates bearing P812S as of 7th April 2024, aggregated by Scorpio assigned lineage.

Supplementary Table 2 – half maximal effective concentration (EC50) of camostat and E64d

	EC50 (μM)	
**Spike presented on pseudotyped Virus**	**camostat**	**E64d**
WT	16.85	1.99
WT+P812S	23.58	3.24
Δ69/70+D796H	17.69	1.79
Δ69/70+D796H+P812S	23.76	4.50
Delta	19.45	2.11
Delta + P812S	23.59	3.87

Supplementary Table 3 – Geometric mean half-maximal neutralising titre (GMT) of AZD1222 one month post second dose vaccinee sera of P812S mutants

	GMT	
**Spike presented on pseudotyped Virus**	**32 °C**	**37°C**
WT	N/A	339.3
WT+P812S	N/A	347.1

Δ69/70+D796H	603.2	293.2
Δ69/70+D796H+P812S	179.8	221.7
Delta	429.8	199.9
Delta + P812S	116.1	132.3

Geometric mean half-maximal neutralising titre (GMT) of AZD1222 vaccinee sera (one month after two doses) at 32°C and 37°C against WT, WT+P812S, Δ69/70+D796H, Δ69/70+D796H+P812S, Delta, and Detla+P812S.
